# Common and specific activations supporting optic flow processing and navigation as revealed by a meta-analysis of neuroimaging studies

**DOI:** 10.1007/s00429-024-02790-8

**Published:** 2024-04-09

**Authors:** Valentina Sulpizio, Alice Teghil, Sabrina Pitzalis, Maddalena Boccia

**Affiliations:** 1 Department of Psychology, Sapienza University, Rome, Italy; 2 Department of Humanities, Education and Social Sciences, University of Molise, Campobasso, Italy; 3 Department of Cognitive and Motor Rehabilitation and Neuroimaging, Santa Lucia Foundation (IRCCS Fondazione Santa Lucia), Rome, Italy; 4 Department of Movement, Human and Health Sciences, University of Rome ‘‘Foro Italico’’, Rome, Italy

**Keywords:** Spatial navigation, Orientation, Optic flow, Visual motion, fMRI, Egocentric navigation, Precuneus

## Abstract

Optic flow provides useful information in service of spatial navigation. However, whether brain networks supporting these two functions overlap is still unclear. Here we used Activation Likelihood Estimation (ALE) to assess the correspondence between brain correlates of optic flow processing and spatial navigation and their specific neural activations. Since computational and connectivity evidence suggests that visual input from optic flow provides information mainly during egocentric navigation, we further tested the correspondence between brain correlates of optic flow processing and that of both egocentric and allocentric navigation. Optic flow processing shared activation with egocentric (but not allocentric) navigation in the anterior precuneus, suggesting its role in providing information about self-motion, as derived from the analysis of optic flow, in service of egocentric navigation. We further documented that optic flow perception and navigation are partially segregated into two functional and anatomical networks, i.e., the dorsal and the ventromedial networks. Present results point to a dynamic interplay between the dorsal and ventral visual pathways aimed at coordinating visually guided navigation in the environment.

**Supplementary Information:**

The online version contains supplementary material available at 10.1007/s00429-024-02790-8.

## Introduction

Optic flow, i.e., the structured pattern of motion that arises on our retina by the images of objects in the environment as we move through our surroundings (Gibson 1950), is a powerful visual cue we typically use for monitoring the direction and velocity of our movements (self-motion) in the surrounding environment. Thus, perception of optic flow is not only relevant per se but rather is a prerequisite to higher-level functions such as navigation, as we typically rely on the accurate self-motion perception as we navigate through the environment.

Traditionally, optic flow processing and navigation have been ascribed to two distinct neural systems: a dorsal ‘action’ pathway that mediates the on-line processing of visual motion information, likely aimed at monitoring self- to-object spatial relationships to guide goal-directed actions in dynamic visual environments, and a ventral ‘perception’ pathway that mediates the analysis of visual attributes of the visual world to support scene recognition and navigation (Goodale and Milner 1992; Kravitz et al. 2011). Up to now, it is still unclear how visual information carried out in these two partially segregated systems are subsequently integrated into a unified visual percept.

Neurophysiological evidence on monkeys revealed the crucial role of a series of cortical nodes in the analysis of optic flow. For instance, passive viewing of optic flow stimuli activates the parieto-occipital sulcus, likely including V6 (Pitzalis et al. 2021) and area PEc in the anterior precuneus (pCu) (Raffi et al. 2002, 2011). Additionally, the achievement of a robust perception of self-motion has been ascribed to a set of multisensory regions as the dorsal portion of medial superior temporal area (MSTd), the ventral intraparietal area (VIP), the visual posterior sylvian area (VPS) that are particularly implicated in the multimodal estimate of heading by combining visual and vestibular cues to self-motion direction (Duffy 1998; Bremmer et al. 1999; Schlack et al. 2002; Gu 2018; DeAngelis and Angelaki 2012).

As in macaque, optic flow sensitivity of the human brain has been typically studied using coherent visual motion that resembles the continuous changes of optic flow generated by self-motion (i.e., flow fields stimulation, see Pitzalis et al. 2013a, b, c; egomotion compatible vs egomotion incompatible optic flow, see Cardin and Smith 2010). These neuroimaging studies has shown that optic flow processing is implemented in a bilateral circuit with core regions in temporal, parieto-occipital and frontal cortices (Cardin and Smith 2010, Pitzalis et al. 2010, 2013a; Serra et al. 2019; Sulpizio et al. 2020). More specifically, sensitivity to optic flow has been observed in the temporal area MT + (Kolster et al. 2010), in the medial parieto-occipital areas V6 and V6Av (V6 complex or V6 + ; Pitzalis et al. 2006, 2010, 2013b; Cardin and Smith 2010), in the posterior segment of the intraparietal sulcus (pIPS), a location remarkably coincident with the dorsal part of retinotopic area V3A (Tootell et al. 1997; Pitzalis et al. 2010), in the cingulate sulcus visual areas (CSv) and posterior cingulate sulcus area (pCi) (Wall and Smith 2008; Serra et al. 2019), and in two dorsal parietal regions, corresponding to the putative human areas VIP (IPS-mot, Pitzalis et al. 2013c; Bremmer et al. 2001; Sereno and Huang 2006; Cardin and Smith 2010) and PEc (Pitzalis et al. 2019). Also the parieto-insular cortex contains two motion regions, named the parieto-insular vestibular cortex (PIVC) and the posterior insular cortex area (PIC): while the former is a multisensory region, responding to both vestibular and visual stimuli, the latter responds to vestibular stimuli only (Greenlee et al. 2016; Frank et al. 2014; Frank and Greenlee 2018). Details about the acronyms and the anatomical location of the above-described human regions involved in optic flow processing are provided in Table 1.

**Table 1:** Details about the acronyms and the anatomical location of the human regions involved in optic flow processing and human navigation are provided

Label	Area	Anatomical landmark	Anatomical label
Optic flow			
CSv	Cingulate Visual Area	Cingulate sulcus	Cs
PIVC	Parieto-Insular Vestibular Cortex	Parietal operculum	PO
PIC	Posterior Insular Cortex	Retroinsular cortex	RC
VIP	Ventral Intraparietal Area	Superior Parietal Lobule	SPL
pCi	posterior Cingulate area	Precuneus	pCu
PEc	Parietal area E (caudal)	Precuneus	pCu
V6 +	Visual area 6 (complex)	dorsal Parietal-Occipital Sulcus	dPOs
MT +	Middle Temporal area (complex)	Middle temporal Gyrus	MTG
MST	Middle Superior Temporal area	Middle temporal Gyrus	MTG
V3A	Visual area 3A	Superior Occipital Sulcus	SOG
Navigation			
PPA	Parahippocampal Place Area	Parahippocampal gyrus, Fusiform gyrus	PHG, FG
RSC	Retrosplenial Complex	Calcarine Cortex	CC
OPA	Occipital Place Area	Middle Occipital Gyrus	MOG
HC	Hippocampus	Hippocampus	HC
pCu	Precuneus	Precuneus	pCu

Navigation has been extensively studied by neuroimaging in humans. Typically, in these experiments, participants were involved in a series of tasks including navigation in new or familiar environments, wayfinding, reaching and memorizing specific spatial locations, also by using different spatial strategies (i.e., egocentric vs allocentric representations) (see Boccia et al. 2014). Interestingly, an increasing number of navigational studies took advantage of virtual reality to simulate real-world navigation. Although virtual reality has become a popular tool for understanding navigational processes during functional magnetic resonance imaging (fMRI), vestibular inputs are completely abolished in these studies since participants must lie supine and motionless in the fMRI scanner. Notably, it is well established that the vestibular system contributes to spatial signals and navigation, especially by generating head-direction signals in the head-direction cells (Yoder et al. 2014; Cullen and Taube 2017). Thus, the absence of vestibular stimulation and the difference between the body orientation elicited by the virtual environment and that in the real environment might suggest an intrinsic limit of such studies (Taube et al. 2013).

In humans, spatial navigation has been ascribed to a network of areas including ventromedial posterior cortical regions (scene-selective regions), such as the parahippocampal place area (PPA), the retrosplenial complex (RSC), and the occipital place area (OPA). Scene-selective regions encode navigationally relevant visual stimuli such as scenes and buildings (Epstein et al. 1999; Epstein and Higgins 2007; see also Epstein 2008) and play different and complementary roles in human navigation. PPA is mainly involved in representing the local visual scene, in discriminating different views (Park and Chun 2009; Sulpizio et al. 2013, 2014, 2016) and in encoding the spatial significance of landmarks, which is important for real-world navigation (Janzen and van Turennout 2004; Sun et al. 2021). RSC is recruited during real and imagined navigation (Ino et al. 2002; Wolbers and Büchel 2005), retrieval of environment-centered information (Committeri et al. 2004; Galati et al. 2010; Sulpizio et al. 2013, 2016), visuo-spatial mental imagery of familiar environments (Boccia et al. 2015) and encoding of permanent landmarks (Auger et al. 2012; Auger and Maguire 2013). More recently, a few studies have unveiled the role of OPA in spatial cognition, showing that it represents first-perspective motion information in the immediately visible scene (Kamps et al. 2016) and encodes environmental boundaries (Julian et al. 2016) and local navigational affordances (Bonner and Epstein 2017). Beyond the involvement of scene-selective regions, “core” regions of brain network supporting navigation are the hippocampus (HC) and the parietal cortex. A growing number of imaging studies reported the involvement of the HC in spatial navigation and/or map-like representations (Ghaem et al. 1997; Maguire et al. 1998; Wolbers and Büchel 2005; Iaria et al. 2007; Wolbers et al. 2007; Baumann et al. 2010, 2012; Brown et al. 2010, 2012; Morgan et al. 2011; Viard et al. 2011; Baumann and Mattingley 2010; Brown and Stern 2014). Notably, hippocampal “place cells”, i.e., neurons firing at specific positions in space, have been discovered in both freely moving animals (O’Keefe and Dostrovsky 1971), and humans (Ekstrom et al. 2003), supporting the hypothesis that HC contains a metric allocentric representations of the surrounding space (cognitive map) (Aguirre and D’Esposito 1999; Byrne et al. 2007; O’Keefe and Nadel 1978). The parietal cortex, and the pCu in particular, has been considered a critical area supporting egocentric (body-centered) navigation. For example, tasks involving computations of the spatial relationship between the navigator’s heading and a specific goal location (spatial updating) have shown the recruitment of the posterior parietal cortex (Howard et al. 2014; Spiers and Maguire 2006) and pCu (Wolbers et al. 2008). In few words, although allocentric (world centered) and egocentric (body centered) representations are not discrete functions but rather conceptualized as a continuum (Ekstrom et al. 2017), it has been hypothesized that the HC is more implicated in storing metric allocentric representations of space, while the parietal cortex is primary involved in encoding metric egocentric information (Aguirre and D’Esposito 1999; Byrne et al. 2007; O’Keefe and Nadel 1978). Details about the acronyms and the anatomical location of the above-mentioned human regions involved in navigation are provided in Table 1.

Notably, it has been recently suggested a fundamental distinction between rodent and human navigation (Rolls 2023a, b, c; Rolls et al. 2023a, b). Rodent navigation may be based on the place where the rodent is located, with olfactory and somatosensory cues useful for specifying the place where the rodent is currently located, especially during navigation in the dark (see Rolls 2020 for a review). In contrast, given the highly developed visual system, humans and other primates frequently make use of the visual inputs to navigate using distant visual landmarks (Rolls 2021). Thus, while in rodents, navigation is mainly based on a “place” representation, in humans, it mainly relies on a “view” representation, which emphasizes the role of visual cues such as optic flow in guiding navigation.

A series of computational models (Hartley et al. 2000; Raudies et al. 2012; Raudies and Hasselmo 2012; Sherrill et al. 2015) suggest that visual input from optic flow provides information about egocentric (navigator-centered) motion and influences firing patterns in spatially tuned cells during navigation. For example, a computational model by Raudies and coworkers (2012) indicates that optic flow provides information about self-motion to head direction cells to maintain a specific direction and speed along the motion trajectory. Head direction and speed cells drive grid cell responses in the entorhinal cortex that in turn update place cells in the HC (Hasselmo 2009). Alternatively, it has been hypothesized that optic flow can influence border cell activity (Raudies and Hasselmo 2012) that in turn updates place cell responses in the HC (Hartley et al. 2000). It is also well established that head-direction cells integrate visual self-motion cues (i.e., optic flow) with vestibular signals and that this idiothetic information needed to be automatically updated during navigation (Yoder et al. 2014; Cullen and Taube 2017). Overall, these models suggest a link between optic flow processing and navigation although direct evidence of such a link is scarce.

Insights into the existence of a unified system supporting both self-motion processing and visually guided navigation come from a few numbers of studies (Korkmaz Hacialihafiz and Bartels 2015; Schindler and Bartels 2016, 2017; Sulpizio et al. 2020; Cardelli et al. 2023). For example, it has been observed that scene-selective regions (PPA, RSC and OPA) were further modulated by visual motion (Korkmaz Hacialihafiz and Bartels 2015) and that OPA also exhibited a specific response to motion parallax (Schindler and Bartels 2016). Furthermore, an optic flow-dependent modulation of functional connectivity has been found between the early visual cortex and both visual egomotion- and scene-selective areas (Schindler and Bartels 2017). Notably, the cooperation between motion and navigational regions has been documented during goal-directed navigation requiring updating of position and orientation in the first-person (egocentric) perspective (Sherrill et al. 2015).

Taken together, these studies provide evidence of a functional interplay between the cortical pathway specialized in analyzing self-motion compatible optic flow and that supporting spatial navigation. Note, however, that the specific contribution of hippocampal, parietal, and scene-selective regions in processing optic flow information and the role of egomotion regions in spatial navigation as well as the degree to which brain networks supporting the two processes overlap, is still unclear. To address this limitation in the field, a meta-analytic approach can be used to statistically combine the results of studies on optic flow processing and navigation, thus providing mechanistic insight into interactions that might occur between the two processes with a specific focus on the common neural correlates. Our starting-point assumption is that shared activations among these functions would represent the “core” neural substrate deputed to support the ability to keep track of where we are with respect to our environment (spatial updating) since the optic flow has a dominant role in this spatial ability (Cardelli et al. 2023).

We thus used a coordinate-based meta-analysis—namely, activation likelihood estimation (ALE) meta-analysis—to determine both common and specific activations underlying these two domains. To test the hypothesis that they share—at least in part—the same neural substrates, we performed two single ALE meta-analyses on fMRI studies on optic flow processing and spatial navigation and looked at the conjunction between them. We further examined possible similarities (and differences) between optic flow processing and navigation after splitting navigational studies in those relying on egocentric or allocentric navigational strategies. This allowed us to test the hypothesis that optic flow processing and navigation might share common neural substrates, especially during egocentric visual navigation, as supported by computational and imaging evidence (Raudies et al. 2012; Sherrill et al. 2015). Based on these data, we hypothesized a specific role of dorsal regions supporting egocentric (body-centered) navigation and spatial updating, as the pCu, in encoding self-motion-related optic flow and spatial information about one’s position and orientation during navigation.

## Meta-analysis

### Inclusion criteria for papers

An in-depth search was conducted up to November 2022. To be included in the meta-analysis, studies had to meet the following inclusion criteria:

- studies described in peer-reviewed articles using fMRI;
- studies performing a whole-brain analysis (i.e., and articles reporting only results from region of interest (ROI) analyses were thus excluded to avoid inflated significance for these a-priori defined regions).
- studies clearly reporting coordinates of activation foci in a standardized coordinate space (Talairach and Tournoux 1988, or Montreal Neurologic Institute—MNI).
- studies clearly reporting higher activation during optic flow processing and/or spatial navigation compared with a control condition involving similar cognitive and perceptual demands, in order to isolate the neural correlates of these two processes. In this way, we were able to rule out any activation elicited by confounding processes (i.e., visual processing during navigation, and vice versa).
- studies including more than five participants to focus only on robust results.
- studies involving healthy participants aged less than 65 years (as evidenced by the provided age range and/or mean and standard deviation of participants’ age) to exclude any potential confound due to aging related effects (but see the discussion for a potential limitation of the current study).
- studies involving no manipulation of the participants’ psychophysical conditions (e. g., pharmacological manipulations, psychotherapeutic interventions, or other kinds of manipulations), since these manipulations could bias the results.
- studies analyzing the data using univariate approach that revealed localized increased activation (i.e., studies using machine learning and multivoxel pattern analysis were excluded; studies analyzing the data using functional connectivity or related techniques have been discharged as well). The rationale behind this criterion is that these approaches do not always allow isolating the neural correlates of the research issues but rather are used to determine distinguishable patterns of activation elicited by different information (multivoxel pattern analysis) or to establish the patterns of reciprocal connections between these regions (connectivity-based analyses).

**Fig. 1:**
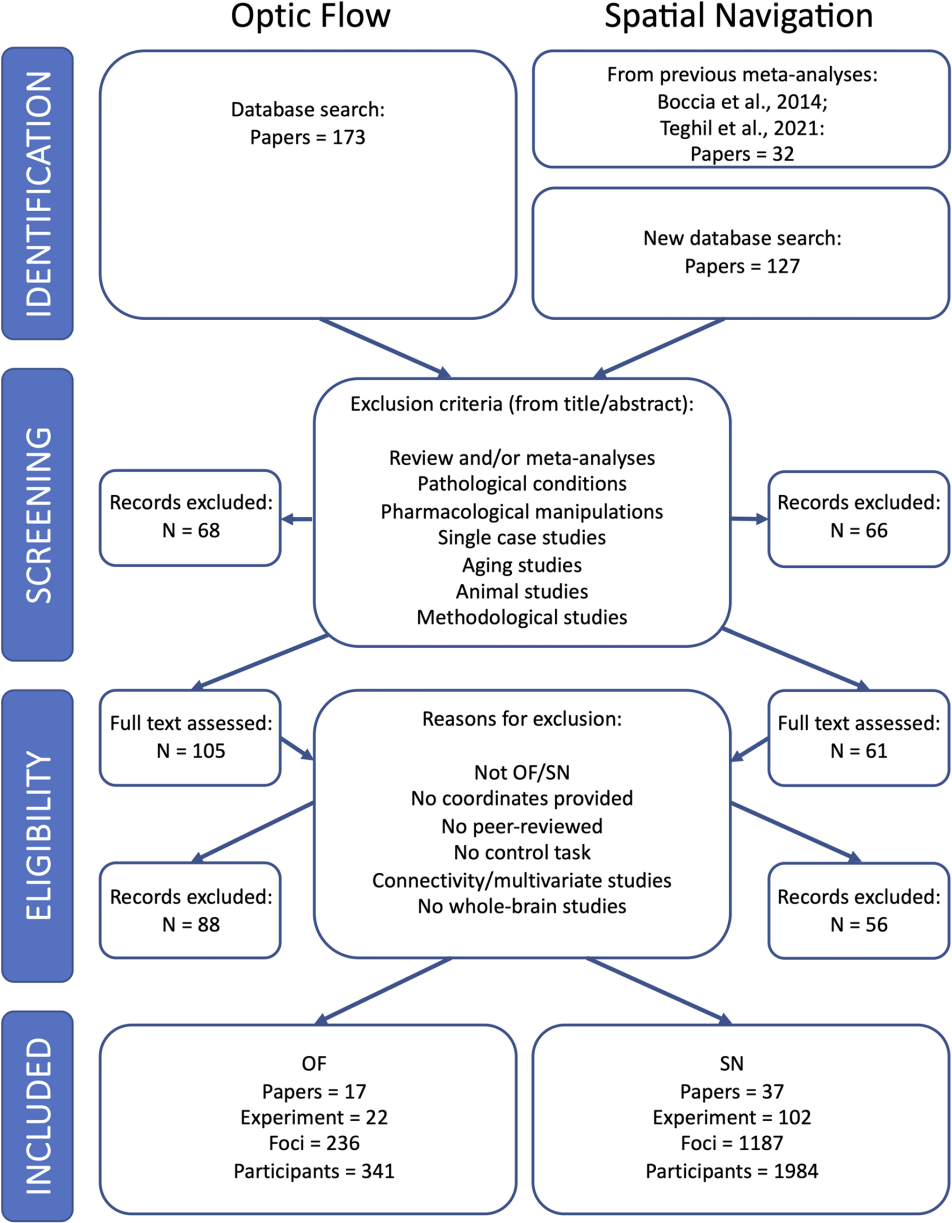
Study selection. PRISMA workflow chart illustrates relevant details about literature selection procedures for the two meta-analyses on optic flow processing (OF) and spatial navigation (SN)

The search was carried out using PubMed, Scopus and ISI. The literature screening and final selection has been performed according to the PRISMA guidelines (Liberati et al. 2009; Moher et al. 2009). This procedure is summarized in the PRISMA flow diagrams (Fig. 1). One author (VS) and a PhD student (TM, in the acknowledgements) extracted and checked the data independently. Two additional authors (AT and MB) double-checked random data and also double-checked data in case of discordance between the first two extractions. Two databases (one for optic flow and one for spatial navigation) were created.

For what concerns the optic flow processing, we performed a systematic literature search with the following string “fMRI AND Optic flow” to include all the experiments testing the neural bases of the visual processing of the optic flow associated to self-motion, using any type of visual stimuli (abstract and/or ecological). A total of 173 original articles were identified. Based on the inclusion criteria (see above; see also Fig. 1 for a detailed description of the PRISMA procedure), a total of 17 original articles (22 experiments) were found eligible to be included in the meta-analysis, with a total of 341 participants. The list of contrasts from each article included in the meta-analysis on optic flow is provided in Table S1.

For the spatial navigation meta-analysis, we included all papers and experiments already included in our previous meta-analyses on environmental navigation (Boccia et al. 2014; Teghil et al. 2021). A further systematic literature search was performed using the following string: ‘fMRI AND (“spatial navigation” or “egocentric” or “allocentric”)’. This search produced 127 resulting original articles; based on the inclusion criteria reported above, 11 experiments from 5 of these articles (Ramanoël et al. 2020, 2022; Riemer et al. 2022; Noachtar et al. 2022; Qi et al. 2022) were included. In addition to the 91 experiments from 32 papers already included in Teghil et al. (2021), the general meta-analysis on spatial navigation was thus performed on 102 experiments from 37 papers for a total of 1984 participants. The list of contrasts from each article included in the meta-analysis on spatial navigation is provided in Table S2.

Further information (when available) about the sample characteristics of the selected papers for both optic flow and spatial navigation meta-analyses (number of participants, mean age, SD age, age range, number of males and females) is provided in Table S3.

### Activation likelihood estimation

Recent guidelines for the meta-analysis (Muller et al. 2018) have been used in the current study. For a quantitative assessment of inter study convergence, the ALE method (Eickhoff et al. 2009; Laird et al. 2005; Turkeltaub et al. 2002) has been applied. The peaks of enhanced activation during optic flow processing (or spatial navigation) compared to the control condition were used to generate an ALE map, using the revised ALE algorithm (Turkeltaub et al. 2012) running under Ginger ALE software (http://brainmap.org/ale/) version 3.0.2.

This approach aims at identifying brain areas with a convergence of reported coordinates across experiments that is higher than expected under the null distribution of a random spatial association of results from these experiments.

To investigate the neural activations respectively associated with optic flow processing and spatial navigation, two separate meta-analyses were performed on the activation foci derived from the selected studies. Coordinates of the foci were taken from all the eligible original papers; Talairach coordinates were converted automatically into MNI coordinates using Ginger ALE.

For spatial navigation, we performed two individual ALE analyses in relation to the spatial strategy (egocentric and allocentric) required by the experimental task. Three experimenters (VS, AT, and MB) independently classified all the experiments included in the meta-analysis on spatial navigation. Experiments that could not be classified into egocentric or allocentric navigation (n = 9, see Table S2) were included only in the general meta-analysis. Finally, a series of conjunction and contrast analyses were conducted.

The conjunction analysis allowed us to investigate which brain regions were commonly activated by optic flow processing and spatial navigation and, which areas were commonly recruited by optic flow processing and egocentric (or allocentric) spatial navigation. On the other hand, contrast analyses allowed us to identify brain regions significantly more activated by optic flow processing compared to spatial navigation and vice versa. Separate contrast analyses were used to highlight brain regions significantly more activated by optic flow processing compared to egocentric (or allocentric) navigation and vice versa.

Statistical ALE maps were thresholded using cluster level correction at p < 0.05 (1000 permutation) with a cluster-forming threshold at voxel-level p < 0.001 (uncorrected) (Eickhoff et al. 2016) in line with the recent guidelines for coordinate based meta-analysis (Muller et al. 2018).

## Results

### General meta-analysis on optic flow processing

**Fig. 2:**
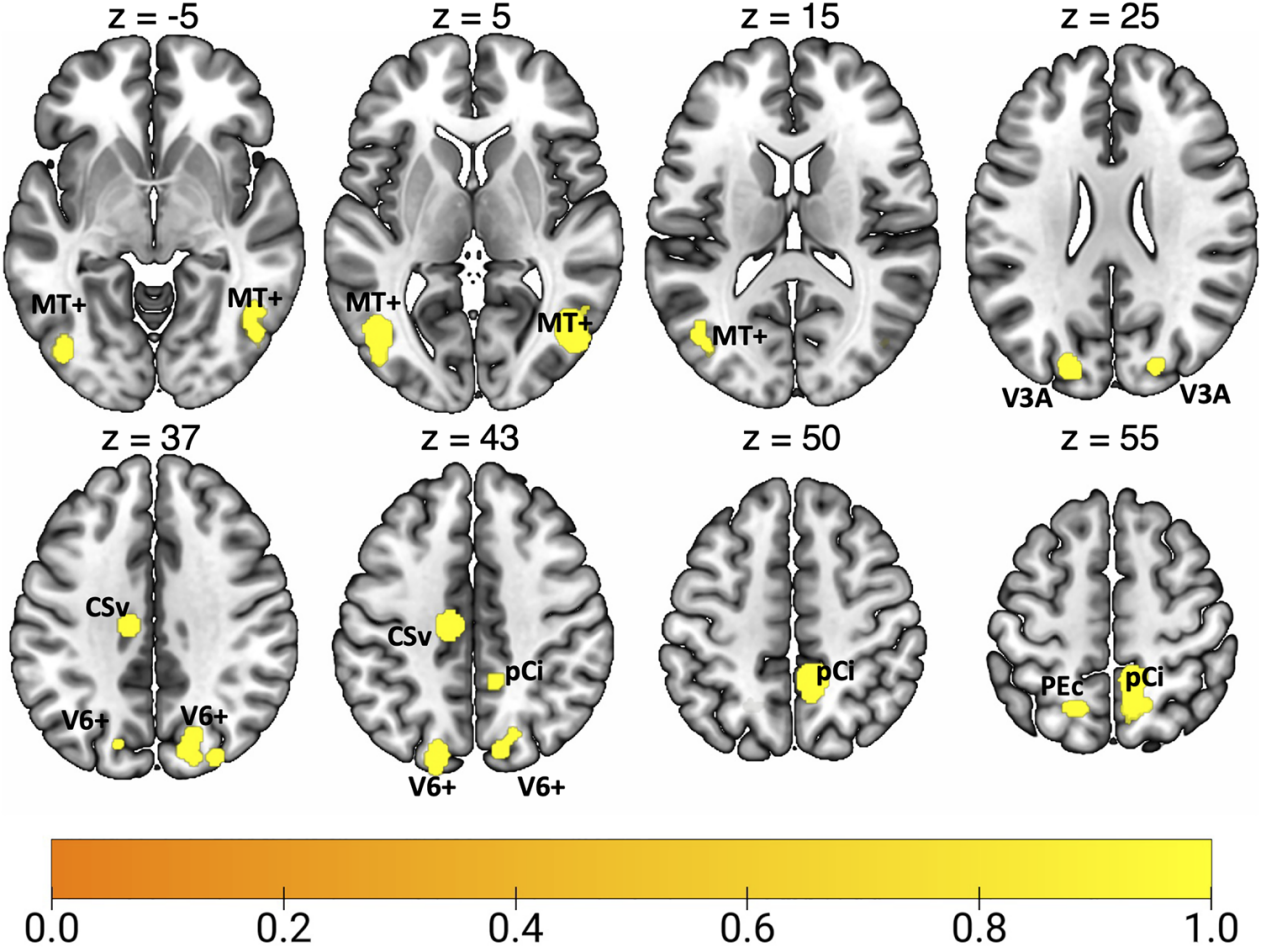
Results of the general ALE meta-analysis on optic flow processing. See Table 1 for the abbreviation meaning of the regional labels

Results of the general ALE meta-analysis on optic flow processing are reported in Fig. 2 and Table 2. This meta-analysis revealed a network of occipital, parietal and frontal regions, encompassing many well-known high-level egomotion regions (as MT+, V3A, V6, IPSmot/VIP, CSv; e.g., Cardin and Smith 2010, Pitzalis et al. 2010, 2013b, c; Serra et al. 2019). A wide cluster of activation was found bilaterally in the middle temporal (MTG) and occipital gyri (MOG), well in correspondence with the motion area MT+ (Kolster et al. 2010; Sulpizio et al. 2022). We also observed bilateral clusters of activation in dorsalmost portion of the parietal occipital sulcus (dPOs), where the motion area V6 is located (Pitzalis et al. 2006, 2010, 2013a; Cardin and Smith 2010), and in the superior occipital gyrus (SOG) close to the pIPS, a location remarkably coincident with the dorsal part of retinotopic area V3A (Tootell et al. 1997; Pitzalis et al. 2010). Moving anteriorly, bilateral foci of activation were found the anterior part of the dorsal pCu, in a region well corresponding to the newly defined human homologue of macaque area PEc. In the left hemisphere, this activation partially included the superior parietal lobule (SPL), likely in correspondence to the human VIP (see Huang and Sereno 2018 for a recent review). In the right hemisphere, the precuneal activation encompassed the pCi, within the posterior dorsal tip of the cingulate sulcus (Cs) (Serra et al. 2019), originally described by Cardin and Smith (2010). In the left hemisphere, a spot of activation was also observed in the depth of the posterior part of the Cs, anterior to the posterior ascending portion of the Cs, corresponding to the original motion area (CSv) described by Wall and Smith (2008).

**Table 2:** Significant activation likelihood clusters for optic flow processing

Cluster	Region	Hemisphere	x	y	z	ALE	Z
1	MOG	RH	46	− 68	4	0.038	7.434
	MTG	RH	54	− 56	2	0.014	3.763
2	MOG	LH	− 44	− 68	6	0.032	6.679
		LH	− 46	− 78	0	0.023	5.425
2	MTG	LH	− 40	− 76	16	0.012	3.400
3	pCu	RH	10	− 46	52	0.033	6.861
		RH	10	− 60	58	0.018	4.486
4	dPOS	RH	20	− 72	40	0.015	4.056
		RH	14	− 80	40	0.015	4.017
	MOG	RH	30	− 82	36	0.013	3.603
	SOG	RH	20	− 84	30	0.018	4.612
5	dPOS	LH	− 18	− 84	44	0.017	4.315
	SOG	LH	− 20	− 86	28	0.017	4.474
6	Cs	LH	− 12	− 20	42	0.031	6.570
7	pCu	LH	− 8	− 58	62	0.012	3.457
	SPL	LH	− 18	− 62	62	0.018	4.634

For each cluster region, label, hemisphere, MNI coordinates, ALE value, and z score are provided. Regions are labeled as followed: *MOG* middle occipital gyrus, *MTG* middle temporal gyrus, *pCu* precuneus, *dPOs* dorsal parieto-occipital gyrus, *SOG* superior occipital gyrus, *Cs* cingulate sulcus, *SPL* superior parietal lobule, *LH *left hemisphere, *RH *right hemisphere

### General meta-analysis on spatial navigation

**Fig. 3:**
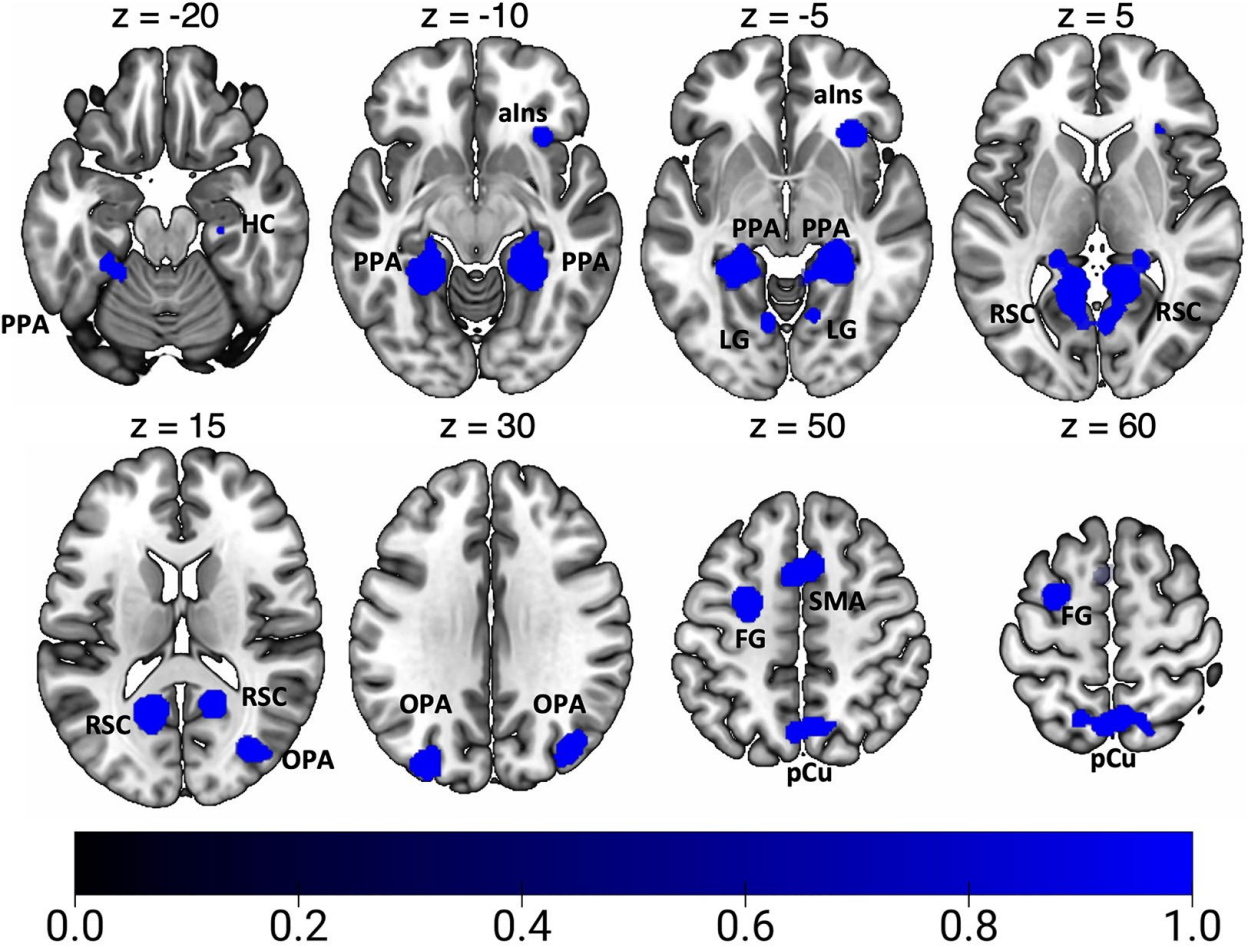
Results of the general ALE meta-analysis on spatial navigation. See Table 1 for the abbreviation meaning of the regional labels

Results of the general ALE meta-analysis on spatial navigation are reported in Fig. 3 and Table 3. In line with previous meta-analyses (Boccia et al. 2014; Cona and Scarpazza 2019; Teghil et al. 2021), we found a bilateral network of areas within the parieto-occipital and the temporo-occipital cortex. In particular, we found bilateral clusters of activation in the ventromedial cortex in correspondence of the parahippocampal gyrus (PHG), lingual gyrus (LG), fusiform gyrus (FG), and HC. This ventromedial activation includes the PPA in the posterior PHG. We also observed a bilateral cluster of activation in the calcarine cortex (CC), at the junction with the ventral portion of the parieto-occipital sulcus (POs), well in correspondence with the scene-selective RSC. Further clusters of activation were found in the pCu, extending into the cortical territory hosting area PEc anteriorly and the area V6Ad posteriorly, and in the adjacent SPL. Additionally, we observed spots of activation in the pIPS, extending into the middle occipital gyrus (MOG), where the scene-selective OPA is typically located. On the right hemisphere we observed a cluster of activation in dorsal portion of POs, in correspondence of the motion area V6 and in the anterior insula (aIns). Other prominent clusters of activation were found in the middle frontal gyrus (MFG) (partially extending into the superior frontal gyrus) of the right hemisphere and in the bilateral supplementary motor area (SMA).

**Table 3:** Significant activation likelihood clusters for spatial navigation

Cluster	Region	Hemisphere	x	y	z	ALE	Z
1	FG	LH	− 24	− 44	− 12	0.085	9.529
1	LG	LH	− 8	− 70	− 2	0.036	4.982
		RH	10	− 46	2	0.061	7.515
		RH	6	− 68	4	0.030	4.329
		RH	10	− 62	6	0.030	4.280
		RH	14	− 64	− 4	0.027	4.006
1	PHG	LH	− 26	− 30	− 24	0.028	4.144
		RH	24	− 38	− 8	0.084	9.455
	HC	RH	28	− 22	− 16	0.026	3.795
1	CC	LH	− 14	− 58	12	0.090	9.984
		RH	16	− 54	14	0.059	7.341
2	dPOs	RH	14	− 78	42	0.027	3.909
		RH	24	− 74	44	0.029	4.213
	MOG	RH	34	− 76	18	0.048	6.262
		RH	44	− 78	10	0.024	3.607
	pCu	LH	− 2	− 64	56	0.045	5.978
		RH	4	− 62	58	0.042	5.617
	SPL	RH	14	− 64	54	0.030	4.313
3	SPL	LH	− 16	− 62	60	0.025	3.669
4	midFG	LH	− 26	− 2	54	0.065	7.867
5	MOG	LH	− 30	− 82	32	0.052	6.688
6	SMA	LH	− 4	10	54	0.049	6.332
		RH	8	18	46	0.029	4.216
7	aIns	RH	32	24	− 4	0.057	7.165

Significant activation likelihood clusters for optic flow processing. For each cluster region, label, hemisphere, MNI coordinates, ALE value, and z score are provided. Regions are labeled as followed: *FG* fusiform gyrus, *LG* lingual gyrus, *PHG* parahippocampal gyrus, *HC* hippocampus, *CC* calcarine cortex, *MFG* middle frontal gyrus, *SMA* supplementary motor area, *aIns* anterior insula. Other labels as in the caption of Table 2

### Meta-analysis on egocentric navigation

**Fig. 4:**
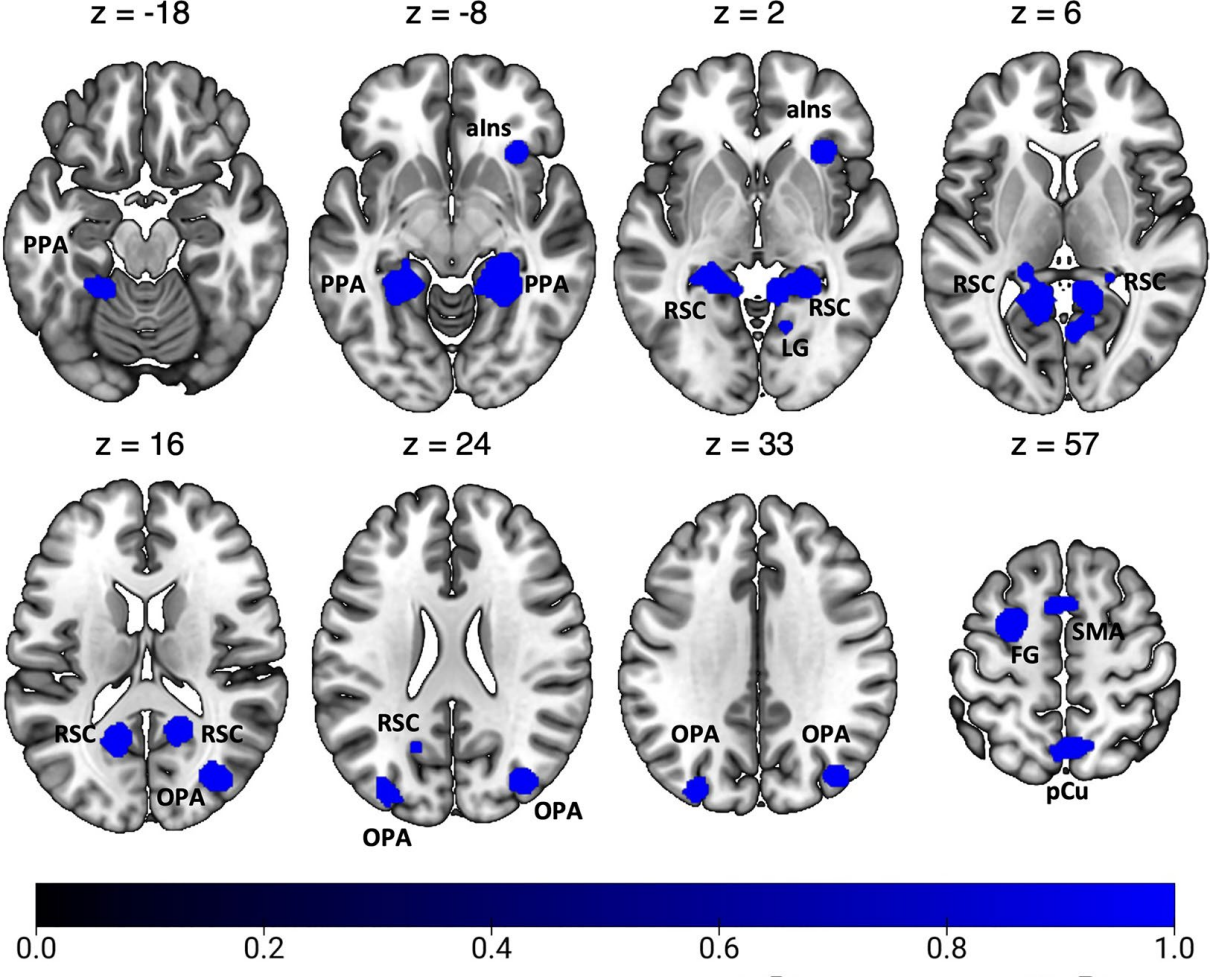
Results of the individual ALE meta-analysis on egocentric navigation. See Table 1 for the abbreviation meaning of the regional labels

Figure 4 and Table 4 show the significant activation clusters related to egocentric navigation. According to previous meta-analyses (Boccia et al. 2014; Cona and Scarpazza 2019; Teghil et al. 2021), we found bilateral foci of activation in the ventromedial cortex in correspondence and around areas PHG (PPA), CC (RSC), HC, and LG. A prominent cluster of activation was found in the left FG. Moving anteriorly, further clusters of activation were found in the pCu, especially in the right hemisphere. This activation likely includes the dorsal portion of the visuomotor areas V6A (V6Ad; Galletti et al. 2022; Tosoni et al. 2015), and PEc (Gamberini et al. 2021; Pitzalis et al. 2019), whose activity has been recently associated in humans with the visuomotor control of navigation and locomotion, respectively (Maltempo et al. 2021). Other bilateral activations were observed in MOG, well in correspondence with the scene-selective OPA. We also observed clusters of activation in the bilateral SMA, in the posterior part of the left superior frontal gyrus and in the right aIns.

**Table 4:** Significant activation likelihood clusters for egocentric navigation

Cluster	Region	Hemisphere	x	y	z	ALE	Z
1	FG	LH	− 22	− 44	− 14	0.050	7.614
	CC	LH	− 14	− 56	12	0.046	7.201
	HC	LH	− 18	− 38	2	0.026	4.881
	PHC	LH	− 22	− 30	− 10	0.015	3.277
2		RH	26	− 40	− 10	0.056	8.230
	LG	RH	10	− 46	2	0.040	6.532
	CC	RH	16	− 54	14	0.038	6.338
	LG	RH	4	− 66	4	0.021	4.058
		RH	10	− 62	6	0.020	4.003
		RH	14	− 62	− 4	0.017	3.554
3	MOG	RH	34	− 76	18	0.035	5.988
	SOG	RH	40	− 76	34	0.027	5.030
	MOG	RH	44	− 78	10	0.016	3.316
4	pCu	RH	4	− 62	58	0.033	5.718
		RH	8	− 66	50	0.024	4.597
	SPL	LH	− 16	− 60	62	0.018	3.651
	pCu	LH	− 4	− 70	52	0.016	3.336
5	MFG	LH	− 26	0	56	0.034	5.854
6	SMA	LH	− 4	10	54	0.029	5.205
		RH	2	10	52	0.024	4.554
7	MOG	LH	− 32	− 86	26	0.026	4.826
8	aIns	RH	32	24	− 4	0.039	6.436

For each cluster region, label, hemisphere, MNI coordinates, ALE value, and z score are provided. Labels as in the caption of Table 3

### Meta-analysis on allocentric navigation

**Fig. 5:**
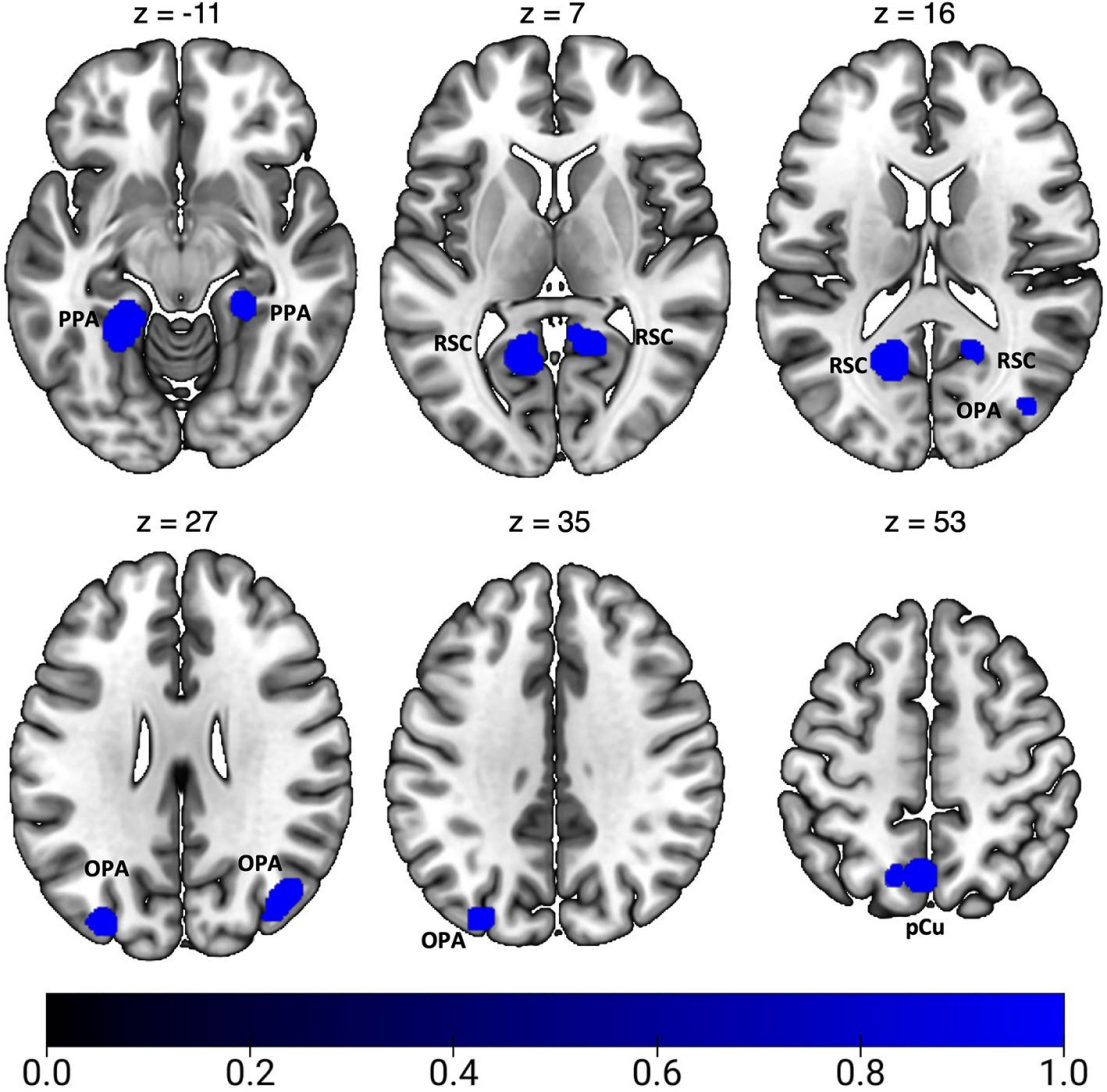
Results of the individual ALE meta-analysis on allocentric navigation. See Table 1 for the abbreviation meaning of the regional labels

Figure 5 and Table 5 show the significant activation clusters related to allocentric navigation. We found that allocentric navigation elicits a set of activations in ventromedial regions, such as the bilateral PHC, FG, CC (RSC) and HC (mainly lateralized in the right hemisphere), as also highlighted by previous meta-analyses (Boccia et al. 2014; Teghil et al. 2021). Another cluster of activation was found in the right vermis (cerebellum). We also found bilateral activations in MOG, well in correspondence with the scene-selective OPA, in the left pCu (in a cortical territory likely including area V6Ad) and in the adjoining SPL.

**Table 5:** Significant activation likelihood clusters for allocentric navigation

Cluster	Region	Hemisphere	x	y	z	ALE	Z
1	PHG/HC	RH	24	− 36	− 8	0.034	5.987
	CC	RH	16	− 52	8	0.026	4.893
		RH	18	− 56	16	0.023	4.448
	vermis	RH	8	− 48	2	0.022	4.381
2	CC	LH	− 16	− 60	14	0.051	7.835
3	FG	LH	− 22	− 44	− 12	0.040	6.656
4	MOG	LH	− 32	− 84	32	0.030	5.382
5	MOG	RH	42	− 74	26	0.023	4.550
		RH	40	− 78	20	0.022	4.287
6	pCu	LH	− 2	− 66	54	0.035	6.086
	SPL	LH	− 14	− 66	52	0.016	3.445
		LH	− 14	− 62	54	0.015	3.223

For each cluster region, label, hemisphere, MNI coordinates, ALE value, and z score are provided. Labels as in the captions of Table 2 and 3

### Conjunction analyses

**Fig. 6:**
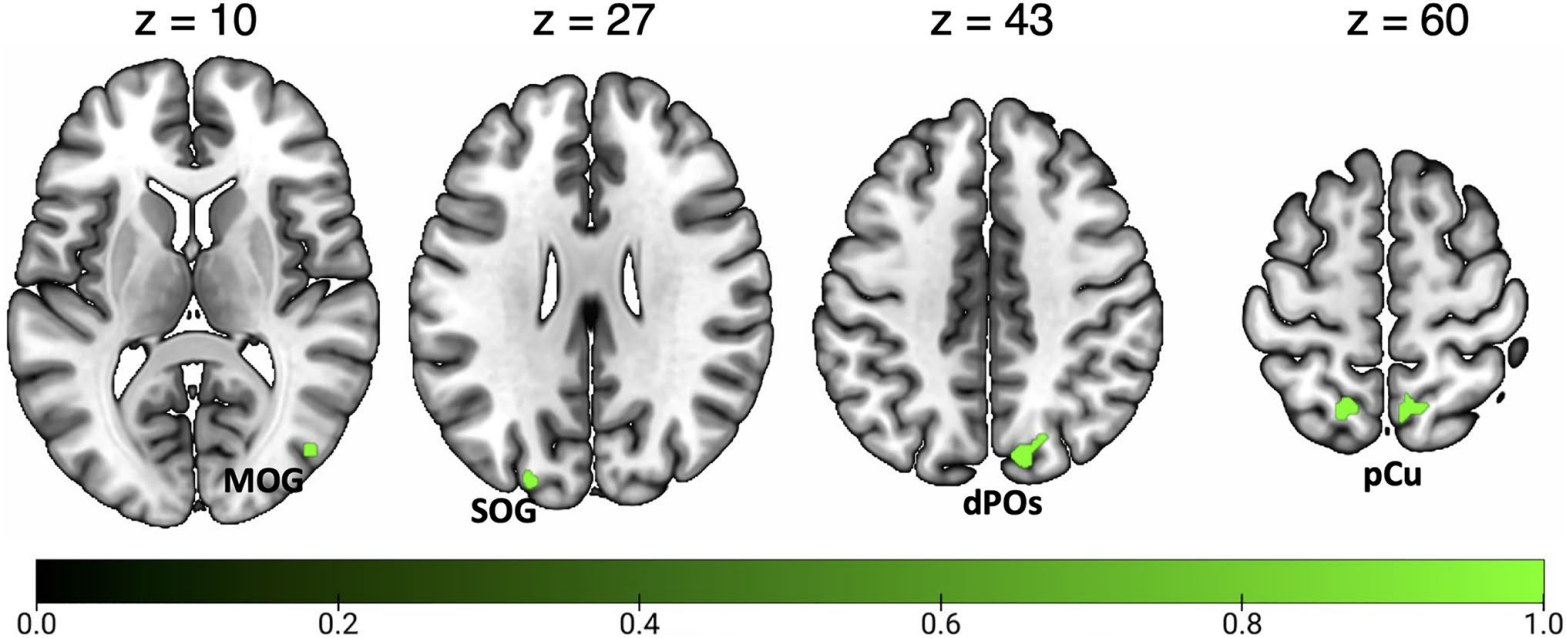
Results of the conjunction analysis between optic flow processing and spatial navigation. See Table 1 for the abbreviation meaning of the regional labels

Figure 6 and Table 6 show the activation clusters commonly activated by optic flow and spatial navigation. This conjunction analysis revealed a pattern of commonly activated regions in the posterior part of the brain. In particular, a prominent focus of activation was found in the bilateral anterior pCu, in a cortical region likely including the newly defined homologue of macaque area PEc. In the left hemisphere, this activation extends into the adjoint SPL. Moving posteriorly, two additional spots of common activation were observed in the dPOs, in correspondence with the motion areas V6 and V6Av. Small foci of common activations were observed in the right MOG and in the left SOG, in a cortical location likely including the retinotopic area V3A.

**Table 6:** Results of the conjunction analyses on optic flow and both general and egocentric navigation

Cluster	Region	Hemisphere	x	y	z	ALE
Optic flow ^ Spatial navigation						
1	pCu	RH	10	− 60	58	0.018
2	SPL/pCu	LH	− 18	− 62	62	0.018
3	dPOs	RH	14	− 80	40	0.015
	pCu	RH	20	− 72	42	0.012
4	MOG	RH	46	− 76	10	0.015
5	SOG	LH	− 22	− 88	26	0.014
6	dPOs	RH	22	− 74	40	0.011
7	pCu	RH	6	− 56	54	0.010
Optic flow ^ Egocentric navigation						
1	pCu	RH	10	− 60	58	0.017
2	pCu/SPL	LH	− 18	− 60	62	0.017
3	MOG	RH	44	− 78	8	0.010
4	SOG	LH	− 22	− 88	26	0.014

For each cluster region, label, hemisphere, MNI coordinates, and ALE value are provided. Labels as in the captions of Table 2 and 3

**Fig. 7:**
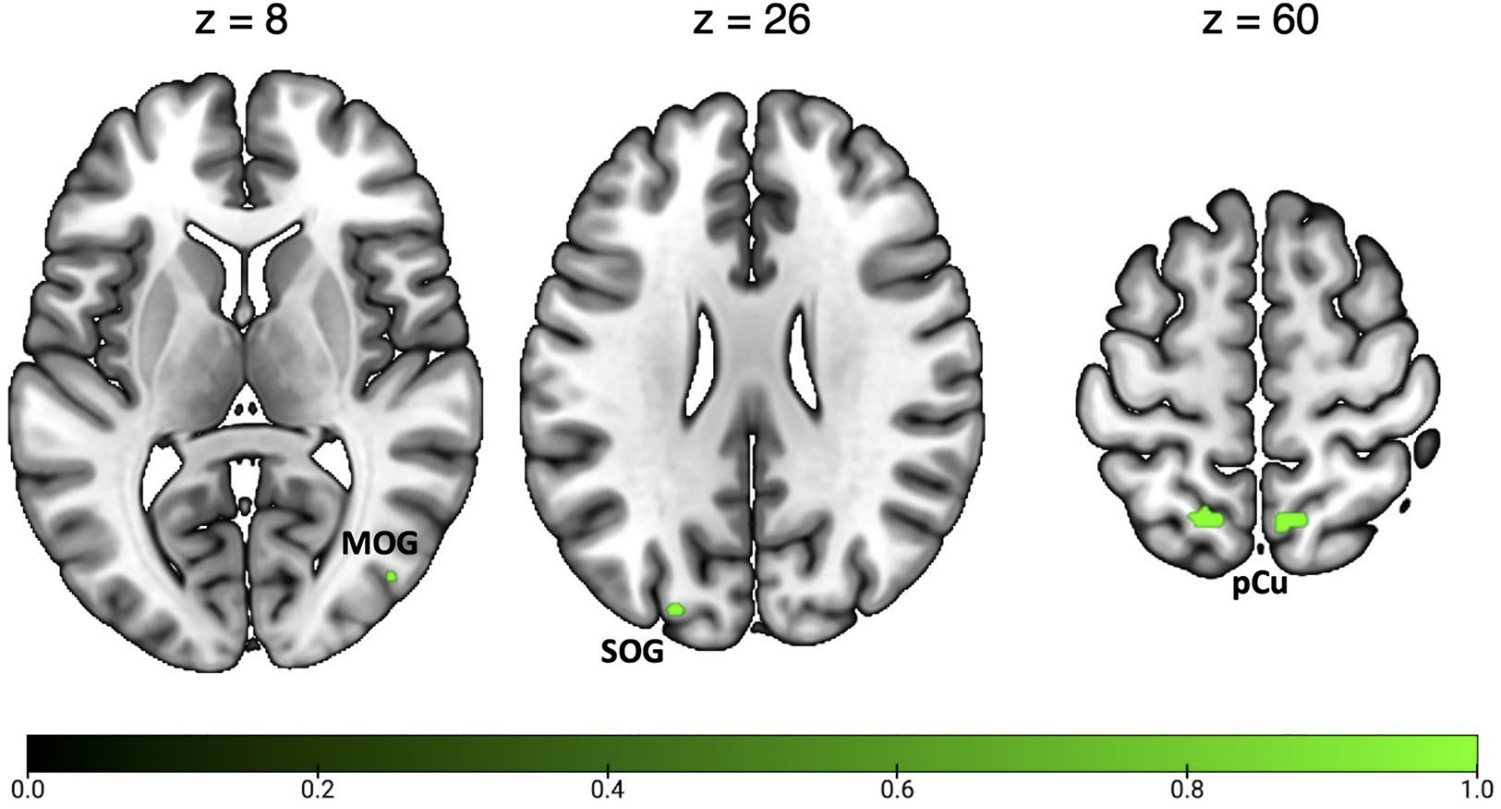
Results of the conjunction analysis between optic flow processing and egocentric navigation. See Table 1 for the abbreviation meaning of the regional labels

Figure 7 and Table 6 show the activation clusters commonly activated by optic flow and egocentric navigation. This conjunction analysis identified common activation in a subset of regions activated by both optic flow and navigation (see above), as the bilateral anterior pCu, the left SOG (including area V3A) and the right MOG.

The conjunction analysis between optic flow and allocentric navigation showed no suprathreshold clusters of activation.

### Contrast analyses

**Fig. 8:**
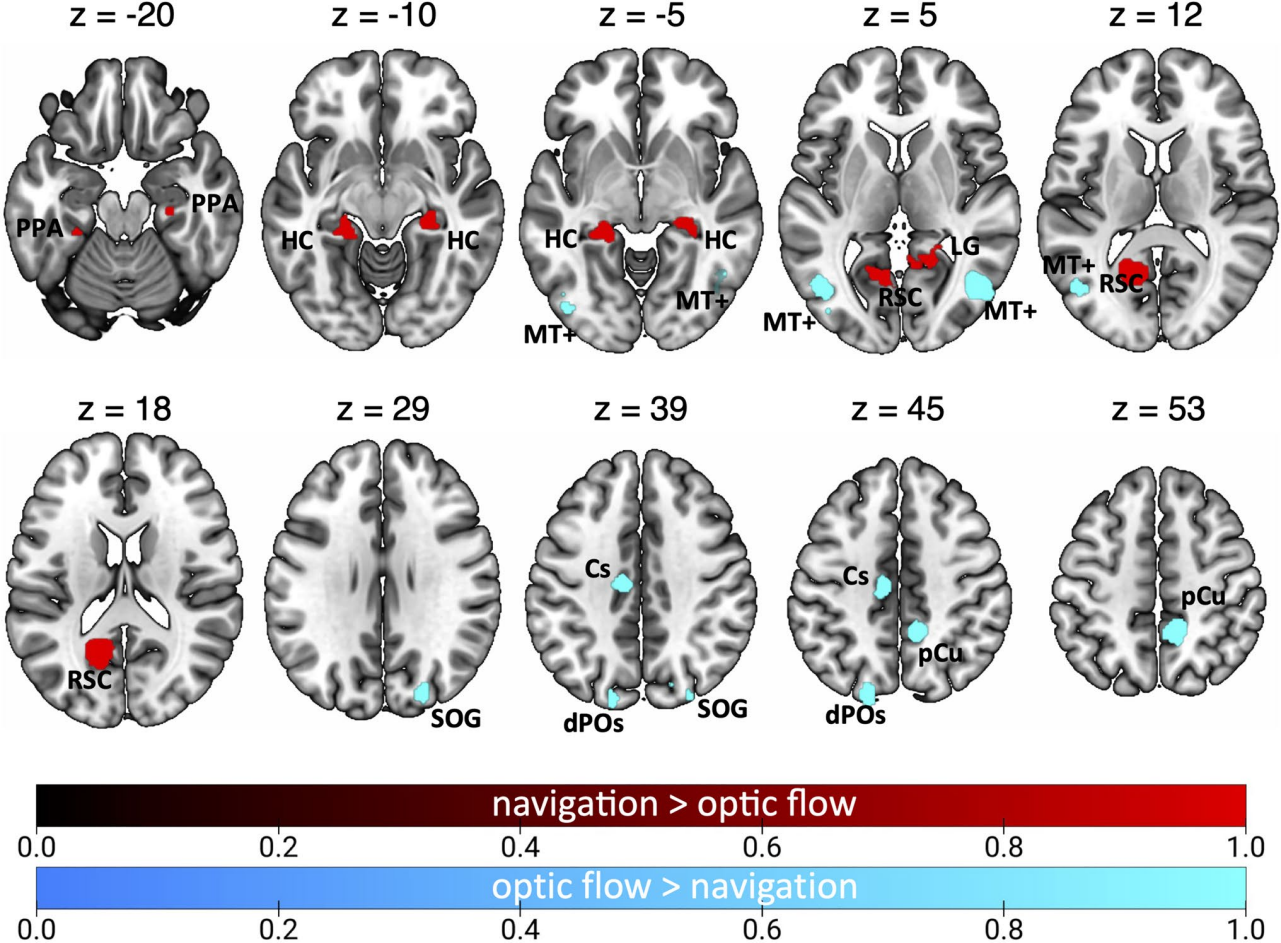
Results of the contrast analysis between optic flow processing and spatial navigation. Brain regions showing higher activation for optic flow processing than spatial navigation are shown in cyan. Brain regions showing the opposite preference (spatial navigation > optic flow) are shown in red. See Table 1 for the abbreviation meaning of the regional labels (color figure online)

Figure 8 and Table 7 show the results of the contrast analyses between optic flow and navigation.

**Table 7:** Results of the contrast analyses on optic flow and spatial navigation

Cluster	Region	Hemisphere	x	y	z	Z
Optic flow > Spatial navigation						
1	MTG	RH	47	− 67	3.4	3.291
2	Cs	RH	11	− 45.9	50.2	3.291
3	MTG	LH	− 46.6	− 68	5.8	3.291
4	Cs	LH	− 9.5	− 19.5	43.2	3.291
		LH	− 16	− 19	38	3.090
5	SOG	RH	24	− 82.9	33.1	3.291
6	dPOs	LH	− 18.2	− 84.9	43.9	3.291
7	MOG	LH	− 42	− 79.7	− 2.7	3.291
	MOG	LH	− 45.6	− 82.9	0.7	3.090
Spatial navigation > Optic flow						
1	CC	LH	− 14	− 60	13	3.090
	CC	LH	− 13	− 57	16	3.291
2	HC	LH	− 23	− 33	− 10	3.291
	PHG	LH	− 24	− 36	− 16	3.090
3	HC	RH	28	− 27	− 11	3.291
	PHG/HC	RH	24	− 32	− 9	3.090
4	CC	RH	18	− 50	3	3.291
	LG	RH	17	− 48	2	3.090

For each cluster region, label, hemisphere, MNI coordinates, and z score are provided. Labels as in the captions of Table 2 and 3

The contrast optic flow > navigation highlighted bilateral clusters in the MTG, well in correspondence with the motion area MT + . We also observed that the bilateral dPOs (likely corresponding to the motion area V6) and the right SOG (likely corresponding to the motion area V3A) are more activated by optic flow as compared to navigation. This contrast also revealed a focus of activation in the left Cs (likely corresponding to the motion area CSv) and in the right pCu, very close the dorsal tip of the Cs (likely corresponding to the motion area pCi).

The contrast navigation > optic flow revealed bilateral clusters in the ventromedial cortex, including the CC in correspondence of area RSC, the PHG (PPA), extending to the HC. This activation also extended into the right LG.

**Fig. 9:**
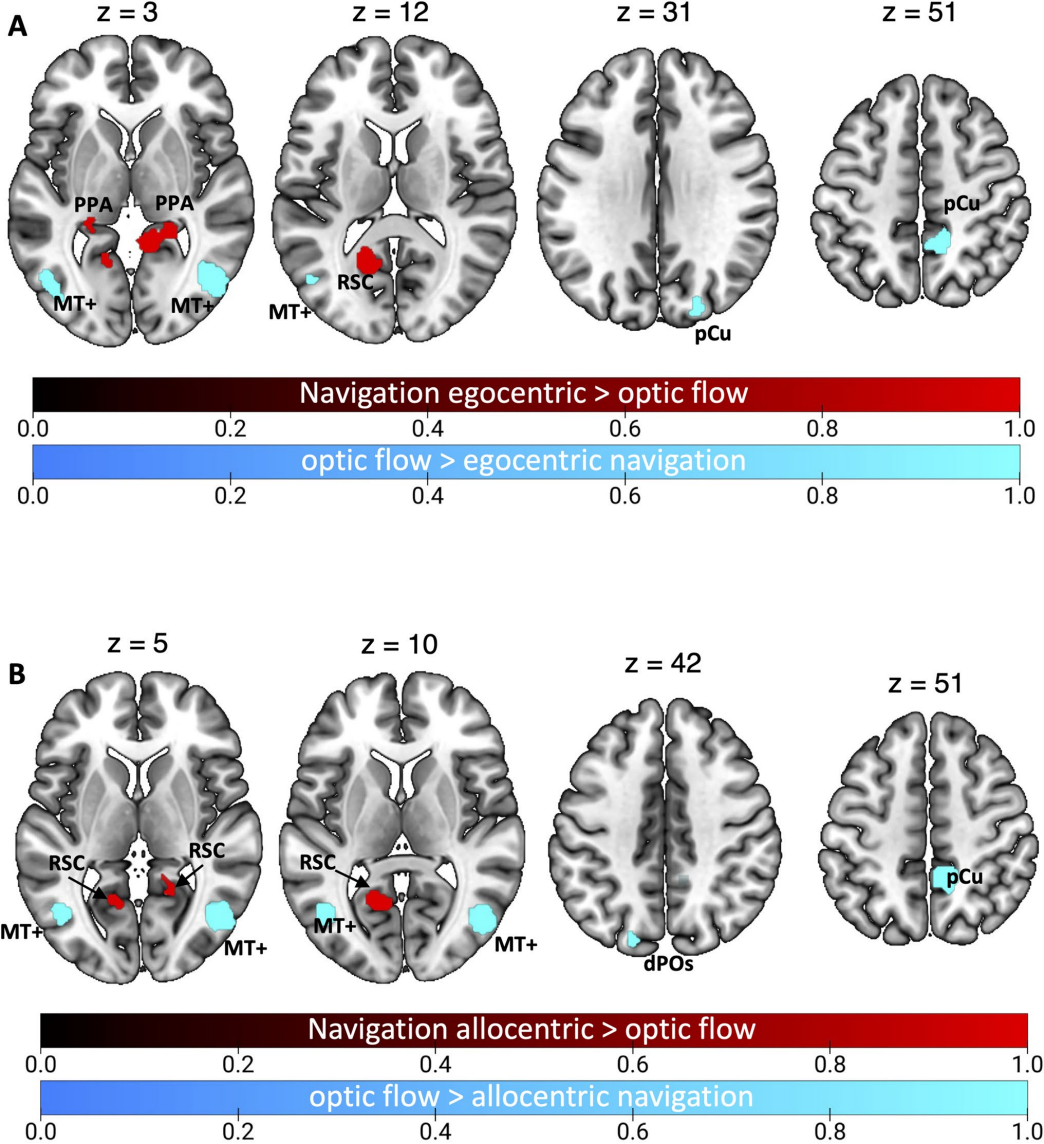
Results of the contrast analysis between optic flow processing and both egocentric (**A**) and allocentric (**B**) navigation. Brain regions showing higher activation for optic flow processing than both egocentric and allocentric navigation are shown in cyan. Brain regions showing the opposite preference (egocentric or allocentric navigation > optic flow) are shown in red. See Table 1 for the abbreviation meaning of the regional labels (color figure online)

After considering the two distinct strategies used in navigation, i.e., egocentric and allocentric, contrast analyses revealed more specific scenarios (see Fig. 9 and Table 8). Figure 9A shows the results of the optic flow vs. egocentric navigation contrast. The contrast optic flow > egocentric navigation (cyan patches) revealed a more prominent involvement of the bilateral MTG, the right pCu (in correspondence of the motion area pCi) and SOG (likely corresponding to the motion area V3A. The opposite contrast (egocentric navigation > optic flow, red patches) revealed the involvement of the bilateral PHG (likely including area PPA) and HC as well as the involvement of the CC/ventral pCu, in correspondence of area RSC. This activation also extended ventrally so that to include part of the vermis (cerebellum) of the left hemisphere.

**Table 8:** Results of the contrast analyses on optic flow and both egocentric and allocentric navigation

Cluster	Region	Hemisphere	x	y	z	Z
Optic flow > egocentric navigation						
1	MTG	RH	48	− 68	2	3.291
2	MTG	LH	− 47	− 71	3	3.291
3	pCu	RH	12	− 47	50	3.291
4	SOG	RH	23	− 85	32	3.291
Optic flow > allocentric navigation						
1	MTG	RH	47	− 68	5	3.291
2	pCu	RH	11	− 46	50	3.291
3	MTG	LH	− 44	− 67	8	3.291
4	dPOS	LH	− 19	− 83	42	3.291
	SOG	LH	− 20	− 78	42	3.090
Egocentric navigation > optic flow						
1	PHG/HC	RH	22	− 41	− 2	3.291
	CC/vermis	RH	9	− 47	2	3.090
2	pCu/CC	LH	− 16	− 59	13	3.291
	pCu/CC	LH	− 14	− 54	16	3.090
3	HC	LH	− 23	− 36	− 5	3.291
	PHG	LH	− 20	− 39	− 4	3.090
Allocentric navigation > optic flow						
1	pCu/CC	LH	− 15	− 60	14	3.291
2	LG	RH	17	− 48	3	3.090
	CC	RH	16	− 54	5	3.090

For each cluster region, label, hemisphere, MNI coordinates, and z score are provided. Labels as in the captions of Table 2 and 3

Figure 9B shows the results of the optic flow vs. allocentric navigation contrast. The contrast optic flow > allocentric navigation (cyan patches) revealed the involvement of the bilateral MTG (including area MT +), the right pCu (including area pCi) and the left dPOs (including area V6). This latter activation extended laterally to include a small portion of SOG (including area V3A).

The opposite contrast (allocentric navigation > optic flow, red patches) showed a bilateral cluster of activation in correspondence of area RSC (at the junction between the ventralmost pCu and CC). In the right hemisphere this activation extended ventrally to include a portion of LG.

## Discussion

Through a systematic ALE meta-analysis, we directly tested whether optic flow processing and spatial navigation share—at least in part—the same neural substrates, as suggested by several studies exploring the functional link between brain areas supporting visual egomotion and scene perception (Korkmaz Hacialihafiz and Bartels 2015; Sulpizio et al. 2020; Schindler and Bartels 2016, 2017). Additionally, the current study aimed at testing the existence of cortical regions commonly recruited by optic flow processing and egocentric navigation, as proposed by computational (Raudies et al. 2012) and experimental studies (Sherrill et al. 2015; Sulpizio et al. 2020).

### Neural correlates of optic flow processing

The results of the general ALE meta-analysis on optic flow processing emphasized the role of a network of posterior cortical regions, including occipital, temporal, parietal and frontal areas.

On the bilateral middle temporal cortex, a prominent focus of activation was observed in correspondence of area MT complex (or MT +), a key motion region of the dorsal visual stream, which retinotopic and functional properties have been widely investigated through the years in electrophysiological, neuropsychological, and neuroimaging studies (Tootell et al. 1995; Morrone et al. 2000; Smith et al. 2006; Kolster et al. 2009; Cardin and Smith 2010). Beyond its general role in processing visual motion, recent evidence suggests that MT + has also a visuomotor role, with the anterior part (corresponding to the anatomical subdivisions FST and MST) responsive to both visual motion and lower-limb movements, suggesting a possible involvement in integrating sensory and motor information to visually guide locomotion (Sulpizio et al. 2022).

A consistent cluster of activation was observed in the bilateral parietal occipital sulcus, in correspondence of area V6, one of the most studied motion areas in the caudal human SPL (see Pitzalis et al. 2013a for a review). Results from several neuroimaging studies revealed that human V6, like macaque V6, is retinotopically organized, responds to unidirectional motion (Pitzalis et al. 2010) and has a strong preference for coherent motion (Cardin and Smith 2010; Helfrich et al. 2013; Pitzalis et al. 2010; von Pföstl et al. 2009). Importantly, area V6 responds to egomotion compatible visual motion, as for example the flow field stimulus (Pitzalis et al. 2010). It is able to distinguish among different types of 3D egomotion (i.e., translational, circular, radial, and spiral motion), with a preference for the translational egomotion (Pitzalis et al. 2013c) and shows, among motion-responsive regions, the highest response bias toward stimuli simulating egomotion in depth (expansion flow) (Pitzalis et al. 2010; Cardin and Smith 2010; Serra et al. 2019), and the highest integration between stereo-depth with 3D motion flow (Cardin and Smith 2011). Further support to the idea that V6 is specifically involved in self-motion perception comes from studies showing that the area responds to changing heading directions (Furlan et al. 2014; Field et al. 2007) and shows a preference for optic flow simulating forward and locomotion-compatible curved paths, indicating its possible involvement in signaling heading changes during locomotion (Di Marco et al. 2021a). V6 is also involved in discounting extraretinal signals (coming from eye and head movements) from retinal visual motion, a neural computation required to infer what is really moving in the scene (Schindler and Bartels 2018a, b; Fischer et al. 2012; Nau et al. 2018) despite concomitant self-motion. This functional property matches with the presence of high percentages of “real-motion” cells in the macaque V6, i.e., cells responsive by the actual movement of an object in the visual field, but not the movement of its retinal image as induced by the eye movements (see Galletti and Fattori 2003 for a review).

In the parieto-occipital surface, immediately posterior to the location of area V6, a more lateral cluster was found to be consistently activated across studies on optic flow processing. This activation falls within the territory of area V3A, a retinotopic area (Tootell et al. 1997) which is typically activated by coherently moving fields of dots simulating the visual stimulation during self-motion (the “flow field” stimulus, Pitzalis et al. 2010). Similarly to V6, it responds to changes of heading directions (Huang et al. 2015; Furlan et al. 2014) and to a visual motion stimulation signaling “real” motion in the visual field (Schindler and Bartels 2018a, b; Fischer et al. 2012; Nau et al. 2018). More recently, it has been proposed that V3A and the adjoining pIPs are specialized in encoding both egomotion- and scene-relevant information, likely for the control of navigation in the surrounding environment (Sulpizio et al. 2020). This area, indeed, is activated by both high- (coherent vs random) and low-level (motion vs static) motion stimulation as well as by navigationally relevant stimuli such as pictures of places (Sulpizio et al. 2020).

Moving anteriorly, optic flow stimulation consistently activates the bilateral pCu. This region likely includes portion of the newly defined human homologue of macaque area PEc (hPEc; Pitzalis et al. 2019). In macaque, this region is involved in visual motion and optic flow processing (Raffi et al. 2002) and integrates information derived from optic flow with somatomotor signals to control and coordinate movements of both upper and lower limbs during the whole-body interaction with the environment (see Gamberini et al. 2020 for a review). Compatibly with this view, the dorsal portion of the anterior precuneus cortex, including area hPEc, is activated during passive observation of both forward and translation egomotion within a virtual environment simulating daily life experiences such as avoiding obstacles while walking (Huang et al. 2015) and by visual motion simulating a change in the self-motion direction (Di Marco et al. 2021a).

In the right hemisphere the activation found in the pCu extends within the posterior segment of the Cs so that likely includes the egomotion area pCi (Serra et al. 2019). Beyond the preference for self-motion compatible optic flow, this area is specifically activated by visual motion simulating a locomotion-compatible curved path, suggesting a role in encoding heading changes and in the estimation of path curvature (Di Marco et al. 2021a). pCi is also activated by a pure motor task requiring participants to perform long-range leg movements (Serra et al. 2019), and exhibits an adaptation effect only when the direction of visually-induced self-motion is compatible with the direction of leg movements, suggesting a role in the multisensory integration of visual and somatomotor cues to guide locomotion (Di Marco et al. 2021b). Compatibly with this view, area pCi has been recently described as more activated by congruent as compared to incongruent combinations of visual and head motion signals, further supporting a role in multimodal self-motion integration (Schindler and Bartels 2018a).

A further focus of activation related to optic flow processing has been observed in the left Cs, well in correspondence of the motion area CSv. This area, originally described by Wall and Smith (2008), is active during visual stimulation but only if that stimulation is indicative of self-motion (Cardin and Smith 2010; Wada et al. 2016). CSv is strongly activated by an optic flow stimulus simulating a curved trajectory (Di Marco et al. 2021a) and by continuous changes in heading directions (Furlan et al. 2014). It is also active during vestibular stimulation (Greenlee et al. 2016; Smith et al. 2012) and connectivity data suggest that it receives proprioceptive input (Smith 2021). CSv, indeed, has strong connectivity with the medial motor areas in both macaques and humans, particularly the cingulate motor areas and SMA (Smith et al. 2018). As pCi, CSv is activated by long-range leg movements (Serra et al. 2019). Taken together, these pieces of evidence support the idea recently proposed by Smith (2021) that CSv acts as a sensorimotor interface for the control of locomotion.

Overall, these findings confirm the existence of a distributed network of cortical regions spanning from the occipital, temporal, and parietal to the frontal cortex specialized in processing optical flow information.

### Neural correlates of spatial navigation

Concerning spatial navigation, the ALE meta-analysis revealed bilateral clusters of activation in the ventromedial cortex encompassing the FG and LG as well as the PHG, including the scene-selective area PPA, the CC in correspondence of the scene-selective RSC and the HC. Further clusters of activations included the bilateral middle occipital sulcus (MOG, in correspondence of the scene-selective OPA), the pCu, the SMA and the left middle frontal gyrus (MFG) and in the right dPOs in a cortical location well corresponding to the retinotopic area V6. Notably, these regions respond to different functions in spatial navigation. For example, PPA is mainly involved in representing the local spatial scene, whereas the RSC is more involved in situating the scene within a larger extended environment (Epstein 2008; Epstein and Higgins 2007; Epstein and Kanwisher 1998; Epstein et al. 2007; Sulpizio et al. 2013, 2016). Additionally, PPA (together with the HC) exhibited more similar multivoxel patterns after learning the object-to-place association, thus indicating a specific role in encoding objects based on their navigational significance (Sun et al. 2021). OPA responds to environmental boundaries (Julian et al. 2016) and local navigational affordances (Bonner and Epstein 2017) and represents first-perspective motion information in the immediately visible scene (Kamps et al. 2016). Notably, OPA partially corresponds to the egomotion area V3A, in agreement with the idea that they are part of a unique motion-selective complex (see also the conjunction analysis) specialized in encoding both scene- and egomotion-related information (Sulpizio et al. 2020). The hippocampal involvement in spatial navigation is well documented. Several neuroimaging studies demonstrated its role in encoding distance to the goal during navigation (Balaguer et al. 2016; Howard et al. 2014; Patai et al. 2019; Sarel et al. 2017), and directional information in large-scale environments (Sulpizio et al. 2018) and in constructing coherent spatial scenes (Maguire et al. 2015). Remarkably, the HC is known to support a map-like spatial representation which reflects metric distances (Morgan et al. 2011; Sulpizio et al. 2014) and landmark positions (Vass and Epstein 2013), similarly to what observed in animals (O’Keefe and Nadel 1978; Hafting et al. 2005).

The role of SMA in spatial navigation is somehow less investigated. This area, and the pre-supplementary motor (pre-SMA) in particular, have been associated to working memory and spatial mental imagery (Nachev et al. 2008; Mellet et al. 2000). Previous studies suggested that pre-SMA is involved in planning and controlling visually guided behavior (Nachev et al. 2008) and in visuo-spatial processing, independently by motor sequence operations (Leek et al. 2016).

Additional clusters of navigation-related activation were observed in correspondence and around the pCu and the dorsal parietal-occipital sulcus (POs). Interestingly, these foci of activation correspond to the motion-related cortical regions hPEc and V6 described as sensitive to egomotion-compatible optic flow. This represents the first descriptive evidence of the current study suggesting common activations for optic flow processing and spatial navigation, as formally demonstrated by the conjunction analysis (see below).

### Common neural activations for optic flow processing and navigation

Optic flow is a powerful visual signal that can be used in several daily activities implying motion such as reaching and/or grasping of moving objects and navigation, since it provides cues that the body is moving in space, which is useful in idiothetic update of spatial representations (Rolls 2023a, b, c).

Although optic flow and navigation might not necessarily involve the same neural systems (note that navigation can be performed in the dark with no optic flow), here we found that a series of brain regions are commonly activated by optic flow processing and spatial navigation. The conjunction analysis between these two domains, indeed, revealed a core neural network including the bilateral pCu, the bilateral middle/superior occipital cortex and the right dorsalmost POs. These cortical regions well correspond to a series of high-level, well known multisensory regions. For example, the pCu activation corresponds to the newly defined human homolog of macaque area PEc (Pitzalis et al. 2019). Recent pieces of evidence suggest that this area is well equipped to process visual motion information to guide body interaction with the external environment. More generally, hPEc is sensitive to two sources of somatomotor and visual stimulations tightly intertwined with the visually guided interaction with the environment, i.e., limb movements (with a preference for leg movements) and egomotion-compatible optic flow. Specifically, hPEc responds to visual motion, as well as to visuomotor and somatomotor tasks requiring lower limb movements (Pitzalis et al. 2019; Maltempo et al. 2021), likely reflecting a role in visually guiding body interaction with the external environment, as during locomotion. More interestingly, it has been demonstrated that hPEc is involved in multisensory integration processes, being able to integrate egomotion-related visual signals with somatomotor inputs coming from leg movements (Di Marco et al. 2021b), likely to guide/adjust leg movements during heading changes. Additionally, besides responding to an abstract pattern of coherent optic flow, especially when it simulates a change in the self-motion direction (Di Marco et al. 2021a), hPEc has a reliable preference for simulated self-motion through a virtual environment (Pitzalis et al. 2020), indicating a stricter sensitivity to visual stimulation reproducing self-displacements in ecologic environments.

Common activation found in the right dorsal POs perfectly matches with the position of the motion area V6 + . This motion area, which includes the two retinotopic areas V6 and V6Av (see Pitzalis et al. 2013a, Sulpizio et al. 2023 for reviews), is typically activated by coherent motion and to egomotion-compatible optic flow (Cardin and Smith 2010; Serra et al. 2019; Pitzalis et al. 2020) by static but navigationally relevant stimuli (Sulpizio et al. 2020), such as images of places (internal and external views of buildings), and it is connected with both PPA and RSC (Tosoni et al. 2015), suggesting that this area may possibly be involved in spatial navigation. Additionally, V6 responds to both visual and auditory cues providing egocentric spatial information useful for navigation (Aggius-Vella et al. 2023).

A small spot of common activation was observed in the superior-occipital sulcus, in proximity of retinotopic area V3A (Tootell et al. 1997). Notably, this area well corresponds to the cortical territory, extending from the pIPS to the border of area V6, which is typically activated by both low-level motion stimulation (contrasting motion vs static, see Sereno et al. 2001; Pitzalis et al. 2010; Sulpizio et al. 2020) and high-level motion stimulation (contrasting coherent vs random motion, see Pitzalis et al. 2010; Serra et al. 2019; Sulpizio et al. 2020). Beside its role in encoding any type of motion information, V3A (as V6) is activated by static but navigationally relevant stimuli (Sulpizio et al. 2020) being partially overlapping with the scene-selective OPA. The direct involvement of V3A (and V6) in navigational tasks has been also suggested by functional connectivity analysis demonstrating a cooperative interaction between these egomotion regions and the navigational responsive regions (HC, retrosplenial cortex, posterior parietal cortex) during goal-direct navigation (Sherrill et al. 2015).

Taken together, these results brought clear evidence of the existence of a common neural network for processing optic flow and navigational information. Since optic flow information is mainly relevant to provide information about self-motion, it seems to be particularly informative during egocentric (first-person) navigation. Crucially, both computational models and experimental evidence support this view suggesting that visual input from optic flow provides information about egocentric but not allocentric (map-based) navigation (Hartley et al. 2000; Raudies et al. 2012; Raudies and Hasselmo 2012; Sherrill et al. 2015). By performing further conjunction analyses between optic flow processing and both egocentric and allocentric navigation we aimed at examining the hypothesis of a specific interplay between optic flow processing and egocentric navigation. Current results confirmed this hypothesis, showing that only the conjunction between optic flow processing and egocentric navigation revealed common foci of activation. Specifically, this analysis highlighted surviving clusters of common activation in the pCu and the superior/middle occipital gyri, indicating as these cortical territories, likely hosting areas hPEc and V3A respectively, represent the crucial hubs that transform egomotion-relevant visual information into an egocentric representation useful for navigation. Present results confirm and further extend previous data, by demonstrating a prominent role of these regions in providing information about the navigator’s movement through the environment to support visually guided navigation. Notably, a prominent neural model that accommodates both human and animal findings (Byrne et al. 2007) suggests that short-term egocentric representations reside in the pCu and are updated there during observer motion. This “parietal window” is especially recruited during spatial updating, i.e., when spatial representations of locations are automatically updated by self-motion. For example, Wolbers and co-workers (Wolbers et al. 2008) observed that the activity in the pCu increased as a function of the number of objects to be remembered, and even more during simulated self-motion as compared with static conditions. Notably, several studies demonstrated a dominant role of optic flow in signaling the observer’s change in direction and location and consequently in the spatial updating ability (Loomis and Beall 1998; Warren et al. 2001; Ellmore and McNaughton 2004; Riecke et al. 2007; Campos et al. 2012; Cardelli et al. 2023). The current meta-analysis further emphasizes the dynamic interplay of self-motion processing with the automatic construction of updated representations and provides new insight into the role of the pCu in supporting visually guided egocentric navigation. Future studies should test the exact contribution of this area in combining and manipulating sensory and spatial information to guide a series of whole-body actions towards the surrounding environment, including locomotion and egocentric navigation.

### Distinct neural activations for optic flow processing and navigation

The current meta-analysis revealed the existence of a functional segregation between optic flow processing and spatial navigation. The direct comparison between these conditions revealed a dorso-ventral gradient, with optic flow activating more dorsal regions (the middle/superior temporal gyrus, the dorsal POs, the anterior Cs), and spatial navigation activating more ventral regions (the HC, the retrosplenial cortex and, the lingual/fusiform/parahippocampal gyri).

This “dorso-ventral” segregation has crucially guided visual neurosciences in the last decades (Macko et al. 1982; Mishkin et al. 1983; Ungerleider and Mishkin 1982). Lesions of the dorsal and ventral streams, in both primates and humans, lead to selective deficits in object vision and spatial vision, respectively, leading to their functional characterization as “What” and “Where” pathways (Kravitz et al. 2011; Macko et al. 1982; Mishkin et al. 1983; Ungerleider and Mishkin 1982). A more detailed view of this functional and anatomical segregation is provided by the current contrast analyses between optic flow and both egocentric and allocentric navigation.

Interestingly, regions more consistently activated during optic flow processing than egocentric navigation are mainly lateralized in the right hemisphere and involved the bilateral middle temporal gyrus (i.e., MT +) and the superior-occipital gyrus (i.e., V3A), posterior cingulate/anterior pCu (i.e., pCi) of the right hemisphere. These areas have been previously shown to provide a pivotal contribution to the perception of optic flow. In particular, both MT + and V3A have been described as potentially involved in the “flow parsing mechanism”, i.e., the capability to extract object-motion information from retinal motion signals by subtracting out the overall optic flow (Rushton and Warren 2005; Warren and Rushton 2009; Sulpizio et al. 2024). For example, Royden and Holloway (2014) demonstrated that a model that uses speed- and direction-tuned units, whose responses are based on the response properties of the macaque MT neurons, can successfully identify the borders of moving objects in a scene through which an observer is moving. Similarly, human V3A seems to contribute to perceptual stability during pursuit eye movements (Fischer et al. 2012) and its activity can differentiate between different self-motion velocities (Nau et al. 2018). Interestingly, “real motion cells” have been found in many regions of the visual stream, including areas MT+ and V3A (for a review, see Galletti and Fattori 2003). A similar pattern of results was observed when comparing optic flow processing and allocentric navigation. The bilateral middle temporal gyrus (i.e., MT+) and the right posterior cingulate/anterior pCu (i.e., pCi) were more consistently activated during optic flow processing than during allocentric navigation, thus confirming their specific involvement in encoding optic flow information. Differently from the optic flow > egocentric navigation contrast, we observed that the left dorsal POs (i.e., V6) was more activated during optic flow processing than during allocentric navigation. This suggests that area V6 is selective for optic flow, but only in comparison with allocentric navigation. Of note, we observed a clear involvement of the right V6 in the overall navigation (see the conjunction between optic flow processing and spatial navigation). Present findings show that the “core” network of optic flow processing mainly comprises prominent motion-sensitive regions, such as MT+, CSv, pCi, V3A, and V6 since they were more activated by optic flow as compared to general navigation. Notice that, while some of them preferred optic flow as compared to both egocentric and allocentric navigation (MT+ and pCi), areas V3A and V6 exhibited a selective preference for optic flow as compared to egocentric and allocentric navigation, respectively.

Regions more activated by egocentric (or allocentric) navigation as compared to optic flow processing included portions of the ventromedial cortex including the scene-selective RSC and PPA and the HC. All these regions are known to have complementary roles in spatial navigation, with PPA and RSC mainly implicated in the identification of places/contexts and in supporting spatial transformations necessary for reorientation, respectively, and the HC mainly involved in supporting a metric spatial representation, especially in large-scale environments (Epstein 2008; Nau et al. 2018; Julian et al. 2018; Sulpizio et al. 2018). Present findings support the existence of a “core” navigational network in the occipito-temporal structures.

Although the meta-analytic approach used in the current study offers the unique opportunity to critically evaluate and statistically combine the results of all relevant data for a given research issue, a series of limitations need to be considered. First, this approach, by pooling studies that are dissimilar in some way, could lead to more heterogeneity and thus less likelihood of finding significant convergence. A major source of heterogeneity observed in the screened papers was the age range of participants (see Table S3), thus some caution is required in interpreting the results. Notably, although aging does not lead to a general decline in visual perception, it could have specific effects on the processing of each optic flow component (Guénot et al. 2023). Similarly, age-related deficits in spatial navigation are evident by the middle decade of life, and these are commonly used to understand the trajectories of healthy aging, paving the way for developing targeted behavioral markers for dementia (Yu et al. 2021). Future meta-analyses might specifically test age-related effects to better summarize the knowledge in these research fields.

## Conclusion

Despite the importance of optic flow during spatial navigation, the functional interplay between cortical regions specialized in processing optic flow and that supporting spatial navigation is still debated. The present study capitalizes on the ALE method of meta-analysis to identify the shared neural activations among visual and spatial functions to reveal the common neural substrate supporting them. The meta-analytic approach was also used to identify the specific neural activations associated with each of these functions.

Beyond the observation that optic flow perception and navigation are partially segregated into two functional and anatomical networks, i.e., the dorsal and the ventromedial networks, respectively, according to the classical neural frameworks of visuospatial processing (Ungerleider and Mishkin 1982; Goodale and Milner 1992), we also documented that they shared common activation in the anterior pCu. Instead, optic flow processing and allocentric map-like navigation were not found to share the same network. This pattern of results seems to fit well with the idea that optic flow provides information about egocentric (but not allocentric) navigation, proposed by both computational (Hasselmo 2009; Raudies et al. 2012) and imaging evidence (Sherrill et al. 2015). Notably, present results are consistent with the idea that the pCu is pivotal for combining information from the senses (e.g., dorsal visual stream) with spatial information (Byrne et al. 2007), likely for the purpose of coordinating visually guided navigation through the environment.

### Supplementary Information

Below is the link to the electronic supplementary material.

Supplementary file1 (DOCX 25 KB)

Supplementary file2 (DOCX 41 KB)

Supplementary file3 (DOCX 26 KB)

## Data Availability

Enquiries about data availability should be directed to the authors.
