# Supplementary material for: Common and specific activations supporting optic flow processing and navigation as revealed by a meta-analysis of neuroimaging studies
Source: Brain Struct Funct. 2024 Apr 9;229(5):1021–45. doi: 10.1007/s00429-024-02790-8 (PMC11147901; doi:10.1007/s00429-024-02790-8)
Supplement: Supplementary file 1 — Supplementary file1 (DOCX 25 KB) [file 429_2024_2790_MOESM1_ESM.docx]

| **Article** | **N° of participants** | **N° of contrasts** | **Contrast** |
| --- | --- | --- | --- |
| Antal et al. 2008 | 10 | 1 | Coherent vs. incoherent visual motion |
| Bartels et al. 2008 | 8 | 1 | Peak coordinates activated by motion |
| Biehl et al. 2017 | 19 | 1 | Moving > static (young group) |
| Billington et al. 2013 | 14 | 2 | HeadingNr > BL |
|  |  |  | HeadingNr > BL |
| Fraedrich et al. 2010 | 29 | 1 | Tunnel-scrambled |
| Fraedrich et al. 2012 | 20 | 1 | Meaningful > Indistinct |
| Kovács et al. 2008 | 12 | 1 | Self-motion vs. object-motion |
| Morrone et al. 2000 | 18 | 2 | Flow motion |
|  |  |  | Translation |
| Pitzalis et al. 2013c | 13 | 1 | Motion > static |
| Pitzalis et al. 2019 | 29 | 1 | Flow fields > Random |
| Pitzalis et al. 2020 | 14 | 1 | All motion conditions vs. static conditions |
| Ricciardi et al. 2007 | 7 | 1 | Optic flow (sighted group) |
| Slobounov et al. 2006 | 12 | 2 | Viewing side walls A–P motion |
|  |  |  | Viewing whole room A–P motion |
| van der Hoorn et al. 2010 | 15 | 3 | Forward wide flow vs. stationary control |
|  |  |  | Reversed wide flow vs. stationary control |
|  |  |  | Forward wide flow vs.reversed wide flow |
| Wada et al. 2016 | 13 | 1 | Coherent motion selective response |
| Wolbers et al. 2008 | 22 | 1 | Experiment I, Spatial updating vs. static |
| Wunderlich et al. 2002 | 12 | 1 | Effect of all motion versus baseline |

**Table S1.** Articles included in the meta-analysis on optic flow. For each paper, details about the number of contrasts from each article and the specific contrasts included in the meta-analysis are reported. The reference list for the articles is provided below.

Antal, A., Baudewig, J., Paulus, W., & Dechent, P. (2008). The posterior cingulate cortex and planum temporale/parietal operculum are activated by coherent visual motion. Visual neuroscience, 25(1), 17–26. <https://doi.org/10.1017/S0952523808080024>

Bartels, A., Zeki, S., & Logothetis, N. K. (2008). Natural vision reveals regional specialization to local motion and to contrast-invariant, global flow in the human brain. Cerebral cortex (New York, N.Y. : 1991), 18(3), 705–717. <https://doi.org/10.1093/cercor/bhm107>

Biehl, S. C., Andersen, M., Waiter, G. D., & Pilz, K. S. (2017). Neural changes related to motion processing in healthy aging. Neurobiology of aging, 57, 162–169. <https://doi.org/10.1016/j.neurobiolaging.2017.05.018>

Billington, J., Wilkie, R. M., & Wann, J. P. (2013). Obstacle avoidance and smooth trajectory control: neural areas highlighted during improved locomotor performance. Frontiers in behavioral neuroscience, 7, 9. <https://doi.org/10.3389/fnbeh.2013.00009>

Fraedrich, E. M., Flanagin, V. L., Duann, J. R., Brandt, T., & Glasauer, S. (2012). Hippocampal involvement in processing of indistinct visual motion stimuli. Journal of cognitive neuroscience, 24(6), 1344–1357. <https://doi.org/10.1162/jocn_a_00226>

Fraedrich, E. M., Glasauer, S., & Flanagin, V. L. (2010). Spatiotemporal phase-scrambling increases visual cortex activity. Neuroreport, 21(8), 596–600. <https://doi.org/10.1097/WNR.0b013e32833a7e2f>

Kovács, G., Raabe, M., & Greenlee, M. W. (2008). Neural correlates of visually induced self-motion illusion in depth. Cerebral cortex (New York, N.Y. : 1991), 18(8), 1779–1787. <https://doi.org/10.1093/cercor/bhm203>

Morrone, M. C., Tosetti, M., Montanaro, D., Fiorentini, A., Cioni, G., & Burr, D. C. (2000). A cortical area that responds specifically to optic flow, revealed by fMRI. Nature neuroscience, 3(12), 1322–1328. <https://doi.org/10.1038/81860>

Pitzalis S, Sdoia S, Bultrini A, Committeri G, Di Russo F, Fattori P, Galati G (2013c) Selectivity to translational egomotion in human brain motion areas. PLoS ONE 8:1–14. https://doi. org/10.1371/journal.pone.0060241

Pitzalis, S., Serra, C., Sulpizio, V., Committeri, G., de Pasquale, F., Fattori, P., Galletti, C., Sepe, R., & Galati, G. (2020). Neural bases of self- and object-motion in a naturalistic vision. Human Brain Mapp. 41, 1084–1111. <https://doi.org/10.1002/hbm.24862>

Pitzalis, S., Serra, C., Sulpizio, V., Di Marco, S., Fattori, P., Galati, G., & Galletti, C. (2019). A putative human homologue of the macaque area PEc. Neuroimage 202, 116092

Ricciardi, E., Vanello, N., Sani, L., Gentili, C., Scilingo, E. P., Landini, L., Guazzelli, M., Bicchi, A., Haxby, J. V., & Pietrini, P. (2007). The effect of visual experience on the development of functional architecture in hMT+. Cerebral cortex (New York, N.Y. : 1991), 17(12), 2933–2939. <https://doi.org/10.1093/cercor/bhm018>

Slobounov, S., Wu, T., Hallett, M., Shibasaki, H., Slobounov, E., & Newell, K. (2006). Neural underpinning of postural responses to visual field motion. Biological psychology, 72(2), 188–197. <https://doi.org/10.1016/j.biopsycho.2005.10.005>

van der Hoorn, A., Beudel, M., & de Jong, B. M. (2010). Interruption of visually perceived forward motion in depth evokes a cortical activation shift from spatial to intentional motor regions. Brain research, 1358, 160–171. <https://doi.org/10.1016/j.brainres.2010.08.050>

Wada, A., Sakano, Y., & Ando, H. (2016). Differential Responses to a Visual Self-Motion Signal in Human Medial Cortical Regions Revealed by Wide-View Stimulation. Frontiers in psychology, 7, 309. <https://doi.org/10.3389/fpsyg.2016.00309>

Wolbers, T., Hegarty, M., Büchel, C., & Loomis, J. M. (2008). Spatial updating: how the brain keeps track of changing object locations during observer motion. Nature neuroscience, 11(10), 1223–1230. <https://doi.org/10.1038/nn.2189>

Wunderlich, G., Marshall, J. C., Amunts, K., Weiss, P. H., Mohlberg, H., Zafiris, O., Zilles, K., & Fink, G. R. (2002). The importance of seeing it coming: a functional magnetic resonance imaging study of motion-in-depth towards the human observer. Neuroscience, 112(3), 535–540
