# Supplementary material for: Common and specific activations supporting optic flow processing and navigation as revealed by a meta-analysis of neuroimaging studies
Source: Brain Struct Funct. 2024 Apr 9;229(5):1021–45. doi: 10.1007/s00429-024-02790-8 (PMC11147901; doi:10.1007/s00429-024-02790-8)
Supplement: Supplementary file 2 — Supplementary file2 (DOCX 41 KB) [file 429_2024_2790_MOESM2_ESM.docx]

| **Article** | **N° of participants** | **N° of contrasts** | **Contrast** | **Category** |  |
| --- | --- | --- | --- | --- | --- |
| Baumann et al. 2010 | 17 | 2 | Summary of fMRI findings for the contrast of experimental and baseline conditions during the encoding phase | EGO |  |
|  |  |  | Summary of fMRI findings for the contrast of the experimental and baseline conditions during the retrieval phase | EGO |  |
| Brown et al. 2010 | 22 | 1 | Significant areas of activation from the overlapping > non-overlapping contrast in the critical hall period | EGO |  |
| Burgess et al. 2001 | 13 | 3 | Regional Activations in the Place–Width Contrast | EGO |  |
|  |  |  | Regional Activations in the Place–Object Contrast | EGO |  |
|  |  |  | Regional Activations in the Place–Person Contrast | EGO |  |
| Burles et al. 2017 | 27 | 2 | Spatial ‘orienting’ | ALLO |  |
|  |  |  | Spatial ‘updating’ | EGO |  |
| Burte et al. 2018 | 45 | 1 | Regions that exhibited a relationship between heading disparity and the hemodynamic response | G |  |
| Chan et al. 2013 | 19 | 2 | Aligned > Misaligned | EGO |  |
|  |  |  | Misaligned > Aligned | EGO |  |
| Chrastil et al. 2015 | 24 | 2 | Regions with increasing activity | G |  |
|  |  |  | Correct > incorrect | G |  |
| Gomez et al. 2014 | 18 | 6 | [A > EU] | ALLO |  |
|  |  |  | [A > ERO] | ALLO |  |
|  |  |  | [A > C] | ALLO |  |
|  |  |  | [EU > A] | EGO |  |
|  |  |  | [EU > ERO] | EGO |  |
|  |  |  | [EU > C] | EGO |  |
| Gron et al. 2000 | 24 | 1 | Group data: anatomical regions, Brodmann's areas (BA) and stereotactic coordinates of the voxels of peak activation | EGO |  |
| Hartley et al. 2003 | 16 | 2 | Between-Subjects W-T | ALLO |  |
|  |  |  | Between-Subjects W-Ro | ALLO |  |
| Hirshhorn et al. 2011 | 13 | 3 | Regions of Common Activation for Distance and Proximity Judgment and Blocked-Route Problem Solving in Session 1 | ALLO |  |
|  |  |  | Regions of Common Activation for Distance and Proximity Judgment and Blocked-Route Problem-Solving in Both Session 1 and Session 2 | ALLO |  |
|  |  |  | Regions of Activation Unique to Session 2 | ALLO |  |
| Iaria et al. 2007 | 9 | 1 | Using of cogntive maps (retrieval vs. control) | ALLO |  |
| Iaria et al. 2008 | 10 | 4 | Neural activity related to the performance of the highly familiar pathway | EGO |  |
|  |  |  | Neural activity related to the direct contrasts between action events and the perceptual event, Event B > Event A | EGO |  |
|  |  |  | Neural activity related to the direct contrasts between action events and the perceptual event, Event C > Event A | EGO |  |
|  |  |  | Neural activity related to the direct contrasts between action events and the perceptual event, Event D > Event A | EGO |  |
| Ino et al. 2002 | 16 | 1 | Activated regions during mental navigation | EGO |  |
| Janzen & Jansen 2010 | 20 | 3 | (D-ND objects) > (ND-ND objects) | EGO |  |
|  |  |  | (D-ND objects) > (ND-ND) | EGO |  |
|  |  |  | (DD-D objects) > (ND-ND objects) | EGO |  |
| Janzen & Weststeijn 2007 | 15 | 2 | Increased brain activity for decision as compared to non-decision point items | EGO |  |
|  |  |  | Increased brain activity for in- as compared to against-route items | EGO |  |
| Jordan et al. 2003 | 10 | 2 | Allocentric > Egocentric condition | ALLO |  |
|  |  |  | Navigation > Control Condition | G |  |
| Latini-Corazzini et al. 2010 | 16 | 2 | Survey versus baseline tasks | ALLO |  |
|  |  |  | Route versus baseline tasks | EGO |  |
| Nemmi et al. 2013 | 19 | 1 | Route task | EGO |  |
| Noachtar et al. 2022 | 72 | 4 | Perspective effect in activation, Allocentric > egocentric | ALLO |  |
|  |  |  | Perspective effect in activation, Egocentric > allocentric | EGO |  |
|  |  |  | Euclidian > landmark | G |  |
|  |  |  | Landmark > euclidian | G |  |
| Ohnishi et al. 2006 | 56 | 1 | Virtual maze vs. control task | ALLO |  |
| Qi et al. 2022 | 39 | 1 | DP > nonDP | EGO |  |
| Ramanoël et al. 2020 | 25 | 1 | Landamark > control (young group) | ALLO |  |
| Ramanoël et al. 2022 | 25 | 4 | [OBJ ∩ GEO ∩ FEAT] | ALLO |  |
|  |  |  | [OBJ ∩ GEO] | ALLO |  |
|  |  |  | [OBJ ∩ FEAT] | ALLO |  |
|  |  |  | [GEO ∩ FEAT] | ALLO |  |
| Rauchs et al. 2008 | 16 | 4 | Common Navigation-Related Network | ALLO |  |
|  |  |  | Natural > Impoverished | ALLO |  |
|  |  |  | Alternate > Natural | ALLO |  |
|  |  |  | Alternate > Impoverished | ALLO |  |
| Riemer et al. 2022 | 24 | 1 | B distance > control | EGO |  |
| Rosenbaum et al. 2004 | 10 | 5 | Proximity | ALLO |  |
|  |  |  | Distance | ALLO |  |
|  |  |  | Blocked route | ALLO |  |
|  |  |  | Sequencing | EGO |  |
|  |  |  | Landamark > baseline | EGO |  |
| Rosenbaum et al. 2007 | 7 | 8 | Task vs. baseline, Proximity | ALLO |  |
|  |  |  | Task vs. baseline, Distance | ALLO |  |
|  |  |  | Task vs. baseline, Blocked | ALLO |  |
|  |  |  | Task vs. baseline, Vector | ALLO |  |
|  |  |  | Task vs. baseline, Landmark recognition | EGO |  |
|  |  |  | Task vs. baseline, Landmark perception | EGO |  |
|  |  |  | Task vs. baseline, Sequence | EGO |  |
|  |  |  | Task vs. baseline, Direct route | EGO |  |
| Schinazi et al. 2010 | 16 | 3 | Building location effect (decision point > non-decision point) (data from entire route) | EGO |  |
|  |  |  | Building location effect (decision point > non-decision point) (West portion) | EGO |  |
|  |  |  | Building location effect (decision point > non-decision point) (East portion) | EGO |  |
| Sherrill et al. 2013 | 18 | 9 | Navigation phase, TPP > Survey | ALLO |  |
|  |  |  | Navigation phase, TPP > FPP | ALLO |  |
|  |  |  | Map presentation phase, TPP > Survey | ALLO |  |
|  |  |  | FPP distance analysis | EGO |  |
|  |  |  | Navigation phase, FPP > Survey | EGO |  |
|  |  |  | Navigation phase, FPP > TPP | EGO |  |
|  |  |  | Map presentation phase, FPP > Survey | EGO |  |
|  |  |  | Map presentation phase, FPP > TPP | EGO |  |
|  |  |  | Successful > Unsuccessful, FPP and TPP navigation phases | GO |  |
| Shine et al. 2016 | 9 | 1 | Non-repeat HD > repeat HD | EGO |  |
| Spiers & Maguire 2006 | 20 | 7 | Customer-driven route planning > coasting events | ALLO |  |
|  |  |  | Spontaneous planning > coasting events | ALLO |  |
|  |  |  | Expectation confirmation > coasting events | ALLO |  |
|  |  |  | Expectation violation > coasting events | ALLO |  |
|  |  |  | Customer-driven route planning > response to customers’ irrelevant statements | ALLO |  |
|  |  |  | Customer-driven route planning (switch to new goal) > customer-driven route planning (initial goal) | ALLO |  |
|  |  |  | Customer-driven route planning (initial goal) > customer-driven route planning (switch to new goal) | EGO |  |
| Viard et al. 2011 | 18 | 1 | Results of the contrast comparing all conditions to the control condition (highlighted gate) | EGO |  |
| Wolbers & Büchel 2005 | 11 | 1 | Main effect of learning | ALLO |  |
| Wolbers et al. 2007 | 16 | 1 | Path integration versus control | EGO |  |
| Xu et al. 2010 | 20 | 7 | Blocked > Line Following | ALLO |  |
|  |  |  | Blocked > Normal | ALLO |  |
|  |  |  | Normal > Line Following | EGO |  |
|  |  |  | Without > Line Following | EGO |  |
|  |  |  | Normal > Without | G |  |
|  |  |  | Normal > Blocked | G |  |
| Zhang et al. 2012 | 16 | 2 | Spatial coordinates of clusters showing activation during the SOP > JRD comparison | ALLO |  |
|  |  |  | Spatial coordinates of clusters showing activation during the JRD > SOP | EGO |  |

**Table S2.** Articles included in the meta-analysis on spatial navigation. For each paper, details about the number of contrasts from each article and the specific contrasts included in the meta-analysis are reported, together with the classification of each contrast in the ALE analyses (egocentric navigation [EGO], allocentric navigation [ALLO], included only in the general meta-analysis on spatial navigation [G]). The reference list for the articles is provided below.

Baumann, O., Chan, E., & Mattingley, J. B. (2010). Dissociable neural circuits for encoding and retrieval of object locations during active navigation in humans. NeuroImage, 49(3), 2816–2825. <https://doi.org/10.1016/j.neuroimage.2009.10.021>

Brown, T. I., Ross, R. S., Keller, J. B., Hasselmo, M. E., & Stern, C. E. (2010). Which way was I going? Contextual retrieval supports the disambiguation of well learned overlapping navigational routes. The Journal of neuroscience: the official journal of the Society for Neuroscience, 30(21), 7414–7422. <https://doi.org/10.1523/JNEUROSCI.6021-09.2010>

Burgess, N., Maguire, E. A., Spiers, H. J., & O'Keefe, J. (2001). A temporoparietal and prefrontal network for retrieving the spatial context of lifelike events. NeuroImage, 14(2), 439–453. <https://doi.org/10.1006/nimg.2001.0806>

Burles, F., Slone, E., & Iaria, G. (2017). Dorso-medial and ventro-lateral functional specialization of the human retrosplenial complex in spatial updating and orienting. Brain structure & function, 222(3), 1481–1493. <https://doi.org/10.1007/s00429-016-1288-8>

Burte, H., Turner, B. O., Miller, M. B., & Hegarty, M. (2018). The Neural Basis of Individual Differences in Directional Sense. Frontiers in human neuroscience, 12, 410. <https://doi.org/10.3389/fnhum.2018.00410>

Chan, E., Baumann, O., Bellgrove, M. A., & Mattingley, J. B. (2013). Extrinsic reference frames modify the neural substrates of object-location representations. Neuropsychologia, 51(5), 781–788. <https://doi.org/10.1016/j.neuropsychologia.2013.02.004>

Chrastil, E. R., Sherrill, K. R., Hasselmo, M. E., & Stern, C. E. (2015). There and Back Again: Hippocampus and Retrosplenial Cortex Track Homing Distance during Human Path Integration. The Journal of neuroscience: the official journal of the Society for Neuroscience, 35(46), 15442–15452. <https://doi.org/10.1523/JNEUROSCI.1209-15.2015>

Gomez, A., Cerles, M., Rousset, S., Rémy, C., & Baciu, M. (2014). Differential hippocampal and retrosplenial involvement in egocentric-updating, rotation, and allocentric processing during online spatial encoding: an fMRI study. Frontiers in human neuroscience, 8, 150. <https://doi.org/10.3389/fnhum.2014.00150>

Grön, G., Wunderlich, A. P., Spitzer, M., Tomczak, R., & Riepe, M. W. (2000). Brain activation during human navigation: gender-different neural networks as substrate of performance. Nature neuroscience, 3(4), 404–408. <https://doi.org/10.1038/73980>

Hartley, T., Maguire, E. A., Spiers, H. J., & Burgess, N. (2003). The well-worn route and the path less traveled: distinct neural bases of route following and wayfinding in humans. Neuron, 37(5), 877–888. <https://doi.org/10.1016/s0896-6273(03)00095-3>

Hirshhorn, M., Grady, C., Rosenbaum, R. S., Winocur, G., & Moscovitch, M. (2012). The hippocampus is involved in mental navigation for a recently learned, but not a highly familiar environment: a longitudinal fMRI study. Hippocampus, 22(4), 842–852. <https://doi.org/10.1002/hipo.20944>

Iaria, G., Chen, J. K., Guariglia, C., Ptito, A., & Petrides, M. (2007). Retrosplenial and hippocampal brain regions in human navigation: complementary functional contributions to the formation and use of cognitive maps. The European journal of neuroscience, 25(3), 890–899. <https://doi.org/10.1111/j.1460-9568.2007.05371.x>

Iaria, G., Fox, C. J., Chen, J. K., Petrides, M., & Barton, J. J. (2008). Detection of unexpected events during spatial navigation in humans: bottom-up attentional system and neural mechanisms. The European journal of neuroscience, 27(4), 1017–1025. <https://doi.org/10.1111/j.1460-9568.2008.06060.x>

Ino, T., Inoue, Y., Kage, M., Hirose, S., Kimura, T., & Fukuyama, H. (2002). Mental navigation in humans is processed in the anterior bank of the parieto-occipital sulcus. Neuroscience letters, 322(3), 182–186. <https://doi.org/10.1016/s0304-3940(02)00019-8>

Janzen, G., & Jansen, C. (2010). A neural wayfinding mechanism adjusts for ambiguous landmark information. NeuroImage, 52(1), 364–370. <https://doi.org/10.1016/j.neuroimage.2010.03.083>

Janzen, G., & Weststeijn, C. G. (2007). Neural representation of object location and route direction: an event-related fMRI study. Brain research, 1165, 116–125. <https://doi.org/10.1016/j.brainres.2007.05.074>

Jordan, K., Schadow, J., Wuestenberg, T., Heinze, H., & Lutz JIncke, C. (2003). Different cortical activations for participants using allocentric or egocentric strategies in a virtual navigation task. Brain Imaging, 1–6. doi: 10.1097/00001756-200401190-00026

Latini-Corazzini, L., Nesa, M. P., Ceccaldi, M., Guedj, E., Thinus-Blanc, C., Cauda, F., D'Agata, F., & Péruch, P. (2010). Route and survey processing of topographical memory during navigation. Psychological research, 74(6), 545–559. <https://doi.org/10.1007/s00426-010-0276-5>

Nemmi, F., Piras, F., Péran, P., Incoccia, C., Sabatini, U., & Guariglia, C. (2013). Landmark sequencing and route knowledge: an fMRI study. Cortex; a journal devoted to the study of the nervous system and behavior, 49(2), 507–519. <https://doi.org/10.1016/j.cortex.2011.11.016>

Noachtar, I., Harris, T. A., Hidalgo-Lopez, E., & Pletzer, B. (2022). Sex and strategy effects on brain activation during a 3D-navigation task. Communications biology, 5(1), 234. <https://doi.org/10.1038/s42003-022-03147-9>

Ohnishi, T., Matsuda, H., Hirakata, M., & Ugawa, Y. (2006). Navigation ability dependent neural activation in the human brain: an fMRI study. Neuroscience research, 55(4), 361–369. <https://doi.org/10.1016/j.neures.2006.04.009>

Qi, Q., Weng, Y., Zheng, S., Wang, S., Liu, S., Huang, Q., & Huang, R. (2022). Task-related connectivity of decision points during spatial navigation in a schematic map. Brain structure & function, 227(5), 1697–1710. <https://doi.org/10.1007/s00429-022-02466-1>

Ramanoël, S., Durteste, M., Bécu, M., Habas, C., & Arleo, A. (2020). Differential Brain Activity in Regions Linked to Visuospatial Processing During Landmark-Based Navigation in Young and Healthy Older Adults. Frontiers in human neuroscience, 14, 552111. <https://doi.org/10.3389/fnhum.2020.552111>

Ramanoël, S., Durteste, M., Bizeul, A., Ozier-Lafontaine, A., Bécu, M., Sahel, J. A., Habas, C., & Arleo, A. (2022). Selective neural coding of object, feature, and geometry spatial cues in humans. Human brain mapping, 43(17), 5281–5295. <https://doi.org/10.1002/hbm.26002>

Rauchs, G., Orban, P., Balteau, E., Schmidt, C., Degueldre, C., Luxen, A., Maquet, P., & Peigneux, P. (2008). Partially segregated neural networks for spatial and contextual memory in virtual navigation. Hippocampus, 18(5), 503–518. <https://doi.org/10.1002/hipo.20411>

Riemer, M., Achtzehn, J., Kuehn, E., & Wolbers, T. (2022). Cross-dimensional interference between time and distance during spatial navigation is mediated by speed representations in intraparietal sulcus and area hMT. NeuroImage, 257, 119336. <https://doi.org/10.1016/j.neuroimage.2022.119336>

Rosenbaum, R. S., Ziegler, M., Winocur, G., Grady, C. L., & Moscovitch, M. (2004). "I have often walked down this street before": fMRI studies on the hippocampus and other structures during mental navigation of an old environment. Hippocampus, 14(7), 826–835. <https://doi.org/10.1002/hipo.10218>

Rosenbaum, R. S., Winocur, G., Grady, C. L., Ziegler, M., & Moscovitch, M. (2007). Memory for familiar environments learned in the remote past: fMRI studies of healthy people and an amnesic person with extensive bilateral hippocampal lesions. Hippocampus, 17(12),1241–1251. doi: 10.1002/hipo.20354

Schinazi, V. R., & Epstein, R. A. (2010). Neural correlates of real-world route learning. NeuroImage, 53(2), 725–735. <https://doi.org/10.1016/j.neuroimage.2010.06.065>

Sherrill, K. R., Erdem, U. M., Ross, R. S., Brown, T. I., Hasselmo, M. E., & Stern, C. E. (2013). Hippocampus and retrosplenial cortex combine path integration signals for successful navigation. The Journal of neuroscience : the official journal of the Society for Neuroscience, 33(49), 19304–19313. <https://doi.org/10.1523/JNEUROSCI.1825-13.2013>

Shine, J. P., Valdés-Herrera, J. P., Hegarty, M., & Wolbers, T. (2016). The Human Retrosplenial Cortex and Thalamus Code Head Direction in a Global Reference Frame. The Journal of neuroscience : the official journal of the Society for Neuroscience, 36(24), 6371–6381. <https://doi.org/10.1523/JNEUROSCI.1268-15.2016>

Spiers, H. J., & Maguire, E. A. (2006). Thoughts, behaviour, and brain dynamics during navigation in the real world. NeuroImage, 31(4), 1826–1840. <https://doi.org/10.1016/j.neuroimage.2006.01.037>

Viard, A., Doeller, C. F., Hartley, T., Bird, C. M., & Burgess, N. (2011). Anterior hippocampus and goal-directed spatial decision making. The Journal of neuroscience : the official journal of the Society for Neuroscience, 31(12), 4613–4621. <https://doi.org/10.1523/JNEUROSCI.4640-10.2011>

Wolbers, T., & Büchel, C. (2005). Dissociable retrosplenial and hippocampal contributions to successful formation of survey representations. The Journal of neuroscience : the official journal of the Society for Neuroscience, 25(13), 3333–3340. <https://doi.org/10.1523/JNEUROSCI.4705-04.2005>

Wolbers, T., Wiener, J. M., Mallot, H. A., & Büchel, C. (2007). Differential recruitment of the hippocampus, medial prefrontal cortex, and the human motion complex during path integration in humans. The Journal of neuroscience : the official journal of the Society for Neuroscience, 27(35), 9408–9416. <https://doi.org/10.1523/JNEUROSCI.2146-07.2007>

Xu, J., Evensmoen, H. R., Lehn, H., Pintzka, C. W., & Håberg, A. K. (2010). Persistent posterior and transient anterior medial temporal lobe activity during navigation. NeuroImage, 52(4), 1654–1666. <https://doi.org/10.1016/j.neuroimage.2010.05.074>

Zhang, H., Copara, M., & Ekstrom, A. D. (2012). Differential recruitment of brain networks following route and cartographic map learning of spatial environments. PloS one, 7(9), e44886. <https://doi.org/10.1371/journal.pone.0044886>
