# Supplementary material for: Common and specific activations supporting optic flow processing and navigation as revealed by a meta-analysis of neuroimaging studies
Source: Brain Struct Funct. 2024 Apr 9;229(5):1021–45. doi: 10.1007/s00429-024-02790-8 (PMC11147901; doi:10.1007/s00429-024-02790-8)
Supplement: Supplementary file 3 — Supplementary file3 (DOCX 26 KB) [file 429_2024_2790_MOESM3_ESM.docx]

| Topic | Study | N | age (mean) | age (DS) | range min | range max | males | females |
| --- | --- | --- | --- | --- | --- | --- | --- | --- |
| Optic Flow | Antal et al. 2008 | 10 | 26.80 | 4.40 | 23 | 38 | 3 | 7 |
| Optic Flow | Bartels et al. 2008 | 8 |  |  | 24 | 38 | 3 | 5 |
| Optic Flow | Biehl et al. 2017 | 19 | 23.30 |  |  |  |  |  |
| Optic Flow | Billinton et al. 2013 | 14 | 28.31 | 4.51 | 20 | 36 | 10 | 4 |
| Optic Flow | Fraedrich et al. 2010 | 29 | 25.00 |  |  |  | 17 | 12 |
| Optic Flow | Fraedrich et al. 2012 | 20 | 25.5 | 4.85 |  |  | 8 | 12 |
| Optic Flow | Kovacs et al. 2008 | 12 | 22 |  |  |  | 16 | 6 |
| Optic Flow | Morrone et al. 2000 | 18 |  |  |  |  | 12 | 6 |
| Optic Flow | Pitzalis et al. 2013 | 13 | 26.4 |  | 21 | 36 | 6 | 6 |
| Optic Flow | Pitzalis et al. 2019 | 29 | 27.00 | 5.50 |  |  | 13 | 16 |
| Optic Flow | Pitzalis et al. 2020 | 14 | 24.00 |  | 20 | 26 | 8 | 6 |
| Optic Flow | Ricciardi et al. 2007 | 7 | 27.00 | 2.00 |  |  | 5 | 2 |
| Optic Flow | Slobounov et al. 2006 | 12 |  |  | 21 | 25 | 6 | 6 |
| Optic Flow | van der Hoorn et al. 2010 | 15 | 23.00 | 4.20 |  |  | 8 | 8 |
| Optic Flow | Wada et al. 2016 | 13 | 34.5 |  | 20 | 44 | 7 | 6 |
| Optic Flow | Wolbers et al. 2008 | 22 |  |  | 20 | 30 | 22 | 0 |
| Optic Flow | Wunderlich et al. 2002 | 12 | 28.00 | 7.00 |  |  | 6 | 6 |
| Navigation | Baumann et al. 2010 | 17 | 31.70 | 7.40 |  |  | 17 | 0 |
| Navigation | Brown et al. 2010 | 22 | 21.36 | 3.43 | 19 | 31 | 9 | 13 |
| Navigation | Burgess et al. 2001 | 13 | 27.20 | 1.00 |  |  | 13 | 0 |
| Navigation | Burles et al. 2017 | 27 | 25.38 | 3.03 |  |  | 16 | 11 |
| Navigation | Burte et al. 2018 | 45 |  |  | 18 | 35 | 22 | 23 |
| Navigation | Chan et al. 2013 | 19 | 22 |  | 17 | 31 | 5 | 12 |
| Navigation | Chrastil et al. 2015 | 24 | 23.50 | 4.81 |  |  | 11 | 13 |
| Navigation | Gomez et al. 2014 | 18 | 23.50 | 2.50 | 17 | 30 | 13 | 5 |
| Navigation | Gron et al. 2000 | 24 | 26.05 | 2.05 |  |  | 12 | 12 |
| Navigation | Hartley et al. 2003 | 16 | 28.90 |  | 23 | 40.3 | 16 | 0 |
| Navigation | Hirshhorn et al. 2011 | 13 | 26.70 | 4.00 |  |  | 5 | 8 |
| Navigation | Iaria et al. 2007 | 9 | 24.90 | 4.10 |  |  | 4 | 5 |
| Navigation | Iaria et al. 2008 | 10 | 23.80 |  | 22 | 27 | 5 | 5 |
| Navigation | Ino et al. 2002 | 16 | 32.30 | 6.80 | 20 | 42 | 16 | 0 |
| Navigation | Janzen & Jansen 2010 | 20 | 24.75 |  | 21 | 30 | 10 | 10 |
| Navigation | Janzen & Weststeijn 2007 | 15 | 22.60 |  | 19 | 31 | 8 | 7 |
| Navigation | Jordan et al. 2003 | 10 |  |  | 19 | 27 | 10 | 0 |
| Navigation | Latini-Corazzini et al. 2010 | 16 | 21.20 | 6.60 | 19 | 34 | 16 | 0 |
| Navigation | Nemmi et al. 2013 | 19 | 25.05 | 2.88 |  |  | 11 | 8 |
| Navigation | Noachtar et al. 2022 | 72 | 26.11 | 3.85 | 20 | 34 | 36 | 36 |
| Navigation | Ohnishi et al. 2006 | 56 | 27.48 | 6.24 |  |  | 28 | 28 |
| Navigation | Qi et al. 2022 | 39 | 20.70 | 2.20 |  |  | 24 | 15 |
| Navigation | Ramanoël et al. 2020 | 25 | 25.40 | 2.70 |  |  | 18 | 7 |
| Navigation | Ramanoël et al. 2022 | 25 | 25.40 | 2.70 | 22 | 32 | 18 | 7 |
| Navigation | Rauchs et al. 2008 | 16 | 22.10 |  | 18 | 30 | 8 | 8 |
| Navigation | Riemer et al. 2022 | 25 | 24.90 |  | 20 | 41 | 11 | 14 |
| Navigation | Rosenbaum et al. 2004 | 10 | 26.40 |  |  |  | 10 | 0 |
| Navigation | Rosenbaum et al. 2007 | 7 | 46.57 |  | 44 | 50 | 4 | 3 |
| Navigation | Schinazi et al. 2010 | 16 | 21.00 |  | 19 | 31 | 8 | 8 |
| Navigation | Sherrill et al. 2013 | 18 | 22.80 | 3.50 |  |  | 12 | 6 |
| Navigation | Shine et al. 2016 | 9 |  |  | 19 | 32 | 7 | 2 |
| Navigation | Spiers & Maguire 2006 | 20 | 49.80 | 8.50 | 27 | 59 | 20 | 0 |
| Navigation | Viard et al. 2011 | 18 | 23.33 | 3.96 |  |  | 18 | 0 |
| Navigation | Wolbers & Büchel 2005 | 11 |  |  | 19 | 28 | 11 | 0 |
| Navigation | Wolbers et al. 2007 | 13 |  |  | 21 | 29 | 7 | 6 |
| Navigation | Xu et al. 2010 | 20 | 24.20 |  | 21 | 30 | 20 | 0 |
| Navigation | Zhang et al. 2012 | 16 |  |  |  |  | 8 | 8 |

Table S3. Sample characteristics. For each paper included in both the meta-analyses, details (when available) about number of participants, mean age, SD age, age range, number of males and females are provided.
